# Antibiofilm properties of 4-hydroxy-3-methyl-2-alkenylquinoline, a novel *Burkholderia*-derived alkaloid

**DOI:** 10.1128/msphere.01081-24

**Published:** 2025-05-08

**Authors:** McKinley D. Williams, Taylor R. Sweeney, Sabrina Trieu, Ravi Orugunty, Abdelahhad Barbour, Fereshteh Younesi, Michael Glogauer, Nopakorn Hansanant, Ronald Shin, Shi-En Lu, Kevin Cao, Abraham Tenorio, Sigmund J. Haidacher, Anthony M. Haag, Thomas D. Horvath, Leif Smith

**Affiliations:** 1 Department of Biology, Texas A&M University 14736, College Station, Texas, USA; 2 Antimicrobial Division, Sano Chemicals Inc., Bryan, Texas, USA; 3 Faculty of Dentistry, University of Toronto 7938, Toronto, Canada; 4 Central Alabama High-Field NMR Facility, Structural Biology Shared Facility, Cancer Center, University of Alabama at Birmingham, Birmingham, Alabama, USA; 5 Department of Biochemistry, Molecular Biology, Entomology and Plant Pathology, Mississippi State University, Mississippi State, MIssissippi, USA; 6 Department of Pathology and Immunology, Baylor College of Medicine, Houston, Texas, USA; 7 Texas Children’s Microbiome Center, Texas Children’s Hospital, Houston, Texas, USA; 8 Department of Pharmacy Practice and Translational Research, College of Pharmacy, University of Houston, Houston, Texas, USA; Philipps University of Marburg, Marburg, Germany

**Keywords:** biofilm, *Burkholderia*, antibacterial, antimicrobial resistance, metabolite, quinolone

## Abstract

Biofilms are an important colonization mechanism employed by several microbial species to better establish themselves and monopolize the acquisition of resources across different environs. Some bacteria have evolved specialized metabolites that, when secreted, disrupt the formation and stability of biofilms generated by competing heterospecies, providing the producing organism with an ecological advantage. Soil-derived species are probable candidates for the identification of such compounds, given the intense level of competition that occurs within the terrestrial ecosystem. The MS14 strain of *Burkholderia contaminans* isolated from soil in Mississippi has previously been shown to produce antimicrobial compounds like occidiofungin and ornibactin. In this report, we demonstrate that this strain also produces 4-hydroxy-3-methyl-2-alkenylquinoline (HMAQ-7), an alkaloid-based metabolite structurally similar to others produced by *Burkholderia*. HMAQ-7 was isolated and purified in sufficient quantities to enable the elucidation of its covalent structure and the evaluation of its biological effects. The compound was found to possess a unique ability to inhibit biofilm biosynthesis in several species, including opportunistic pathogens like *Staphylococcus haemolyticus* and within saliva-derived multispecies biofilms. HMAQ-7 also demonstrated an ability to modulate additional cellular behaviors in *Bacillus subtilis,* including motility and sporulation, suggesting that this molecule is important to the interspecies dynamics present across many diverse microenvironments.

**IMPORTANCE:**

The present study furthers our understanding of the structural complexity and the biological functions of the 2-alkyl-4(1H)-quinolone metabolites produced by *Burkholderia* spp. Low micromolar concentrations of HMAQ-7′ induced observable bacterial growth morphology differences. The antibiofilm properties of the HMAQ-7′ characterized in this study will promote future investigations into possible biological and applied roles. The ability to alter biofilm formation using HMAQ-7′ may facilitate *Burkholderia* spp. colonization in a multitude of environments, that is, aquatic, soil, and possibly during infection. HMAQ may subvert competition by potential competitor species in natural environments of *Burkholderia* spp. and possibly lung infections of cystic fibrosis patients.

## INTRODUCTION

The *Burkholderia* genus is comprised of several species, some known to cause severe human infections (melioidosis), that is, *Burkholderia pseudomallei* and *B. mallei,* and many others that are entirely non-pathogenic. The Burkholderia cepacia complex (Bcc) is a group of bacteria that exhibit plant growth-promoting effects (1, 2) while also being associated with antibiotic-resistant infections in patients with cystic fibrosis (3). The *Burkholderia* spp. serve as valuable sources for discovering several structurally diverse natural products exhibiting both antimicrobial activities and novel mechanisms of action (4–6). Considering this perspective, the exploration of *Burkholderia*-derived compounds opens an exciting realm of research for innovative antibiotics as well as furthering our understanding of microbial ecophysiology.

One such group of metabolites is the 4-alkyl-quinolones (4AQs). The 4AQs belong to a subclass of small nonpolar metabolites that are characterized by the presence of a heteroaromatic quinoline nucleus group (7). These quinolones belong to a much wider category consisting of both synthetic and natural compounds known as alkaloids. They are noted for their diverse array of biological and pharmacological properties including their antimicrobial, antinematicidal, and anticancer capabilities (8–11). These molecules (and related compounds) exhibit a notable degree of structural modularity, possessing variable alkylation patterns at positions C2 and C3, while the heterocyclic nucleus serves as basic scaffolding (12–14). Historically, these compounds have been associated with the *Pseudomonas* genus, and several recent reports have also documented their importance within the *Burkholderia* genus (14–16). Indeed, the structural diversity that is characteristic of these molecules may very well explain the differences in function and activities that they have come to possess within these two genera.

The natural permutations that occur in 4AQs have interesting functional implications, much of which are yet to be understood. A great deal of what has been learned about these compounds has come out of variants derived from *Pseudomonas aeruginosa* (14). These compounds have displayed a range of functions far beyond their original assessment as antimicrobials, demonstrating critical roles in quorum sensing, interspecies communication, iron chelation, and immunomodulation (15, 17–21). More recently, several species belonging to the *Burkholderia cepacia* complex (Bcc) have been identified as producing a similar panoply of 4AQ molecules and other alkaloids, many of which have apparent antimicrobial activity (17, 22–25). The 2-alkyl-4(1H)-quinolones, that is, 4-hydroxy-3-methyl-2-alkenylquinolines (HMAQs) and their N-oxide derivatives (HMAQNOs), have a notable presence in *Burkholderia* spp., with these compounds exhibiting apparent antimicrobial capacities (26–29). Despite these many discoveries, much of this work has been somewhat limited by nonoptimal methods that produce extracts or compound isolates of unknown or dubious purity. This same research has also been hamstrung by a consistent reliance upon largely qualitative methods of exploring their activity rather than more robust quantitative ones. These experimental deficiencies in characterizing HMAQ bioactivity were raised by Piochon et al. (28)

Piochon et al. synthesized several HMAQs and their N-oxide derivatives (HMAQNOs) with varying carbon lengths for the 2-alkenyl group to overcome limitations in isolating the compounds from bacterial species (28). In general, the study showed that the HMAQs and HMAQNOs generally had low micromolar inhibitory activity against gram-positive bacteria. The synthesized compounds had antifungal activity against *Cryptococcus neoformans,* but no activity against *Candida albicans* (28). Direct structural analysis of naturally occurring 2-alkyl-4(1H)-quinolones produced by *Pseudomonas* or *Burkholderia* species is limited, and few studies have been previously reported (27, 28, 30–37).

Despite some limitations with the isolation and characterization of 4AQs, the recent reports are exciting for a number of reasons. The discovery of 4AQs within the Bcc (24) and the current lack of knowledge about their bioactivity exemplifies the need to characterize their role within these biological systems. Considering that similar molecules in *Pseudomonas* species serve as virulence factors, gaining an understanding of these molecules in Bcc may provide critical insight into their possible role as virulence factors in cystic fibrosis (38). Given the transposable nature of their structural components (39), it is not outside the realm of possibility that AQ synthesizers are adapting these molecules to a diverse array of biological functions or chemical defenses that could be species/niche-specific. In view of the genetic and ecological diversity of species belonging to the Bcc (1, 40–43), it seems probable that 4AQs may possess many more unique functions that are yet to be discovered.

In this study, we have identified one such molecule produced by the Bcc member, *Burkholderia contaminans* MS14 (38). Herein, we endeavored to provide a concrete explication of the compound in terms of its structural nuances and its biological function. The covalent structure of the HMAQ was determined using high-resolution mass spectrometry (HRMS) and one-dimensional (1D) and two-dimensional (2D) nuclear magnetic resonance (NMR). The biological activity of the compound was tested against a panel of clinically and ecologically relevant species. Observations related to viability as well as induced macrophenotypes were evaluated.

## RESULTS

### Isolation and structural characterization of the HMAQ from *Burkholderia contaminans* MS14

**Fig 1:**
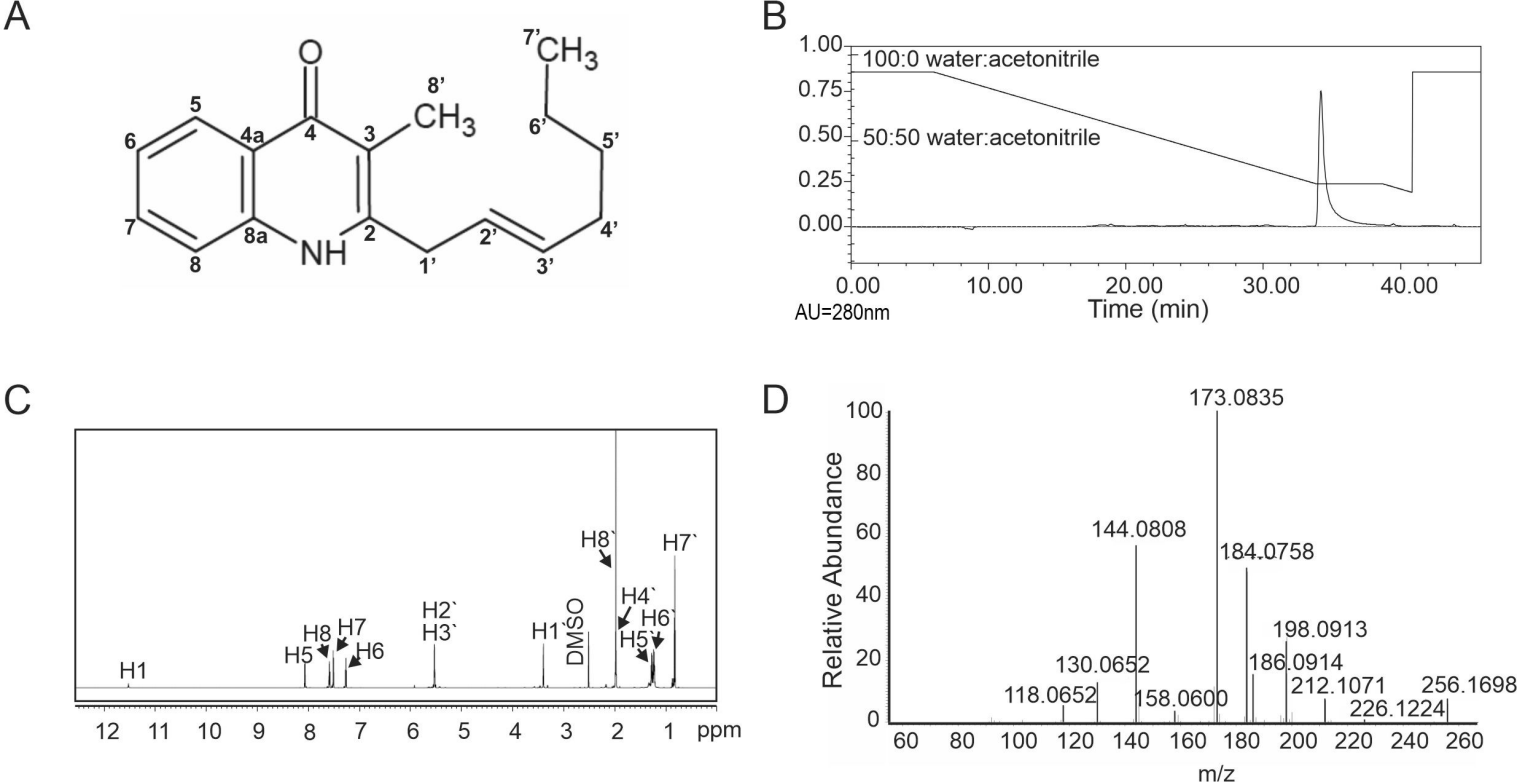
Isolation and structural characterization of the natural product from *B. contaminans* MS14. (**A**) Covalent structure of 4-hydroxy-3-methyl-2-alkenylquinoline (HMAQ) with a seven-carbon alkyl chain (HMAQ-7). The 2′ and 3′ carbons form a *trans* alkenyl group. (**B**) Representative chromatogram of the purified product eluting at 75:25 (acetonitrile:water). (**C**) One-dimensional proton NMR assignment of the product confirming the length of the assigned alkyl chain. (**D**) Tandem HRMSMS spectra for the isolated MS14 product supporting the covalent structure for HMAQ-7.

While isolating the antifungal compound occidiofungin from *B. contaminans* MS14 (44–48), an unknown metabolite was discovered that showed activity against the pathogenic actinomycete *Clavibacter michiganesis*. Structural analysis was subsequently performed, revealing that this inhibitory activity was associated with a novel HMAQ alkaloid (Fig. 1A). The compound has a chemical formula of C_17_H_21_NO, and HRMS analyses identified it as having a protonated molecular ion ([M + H]^+^) of *m/z* 256.1698, which was within 2.0 ppm of the predicted exact mass (*m/z* 256.1623). The unknown compound was separated from a dry culture extract using 100% acetonitrile, which was then purified by high-performance liquid chromatography (HPLC). The nonpolar compound eluted from the preparatory column at a mobile phase composition of approximately 75:25 acetonitrile:water (Fig. 1B). The dry purified compound had an off-white to tan color. Analyses of the 1D proton and carbon NMR data sets and the 2D heteronuclear single quantum coherence (HSQC) and homonuclear multiple bond correlation (HMBC) data sets enabled the deduction of the unknown compound’s covalent structure (Fig. 1A). The metabolite was determined to be an HMAQ compound with a seven-carbon *trans* alkenyl group, referred to as HMAQ-7.

### 1D/2D NMR of HMAQ-7 isolated from *Burkholderia contaminans* MS14

The 1D NMR spectra indicated the presence of aromatic hydrocarbons within the compound. More precisely, H^1^ NMR revealed the presence of one exchangeable amide proton (δ_H_ 11.52) and four deshielded aromatic protons [δ_H_ 8.07(H5), 7.26(H6), 7.59(H7), and 7.51(H8)] (Table 1 and Fig. 1C). The C^13^ data identified nine aromatic carbons including a carbonyl [δ_C_ 148.39(C2), 114.78(C3), 176.94(C4), 123.02(C4a), 125.51(C5), 123.34(C6), 118.09(C7), 131.55(C8), and 139.57(C8a)] (Table 1; Fig. S1). Additionally, the NMR spectra showed the presence of a seven-carbon alkyl chain [δ_C_ 35.33(C1′), 124.78(C2′), 133.34(C3′), 31.89(C4′), 31.31(C5′), 21.97(C6′), and 14.18(C7′)]. A methylene group is present between C2′ and C3′. The J coupling measurements were not discernible in the deuterated dimethyl sulfoxide (DMSO-d6) solvent [5.52(H2′) and 5.53(H3′)]. To measure the coupling value between H2′ and H3′ protons, 1D H^1^ NMR was performed in deuterated chloroform (CDCl3). Evidence of *trans* double bond (J = 15.24) at C-2′/C-3′ was observed (Fig. S2). HMBC data provided evidence for the methylene envelope attached to the C-2 position of the aromatic ring (Table 1; Fig. S3). Finally, one downfield-shifted methyl group was identified as attached to the C3 position of the aromatic nucleus (δ_H_ 1.97(H8′)). Analyses of the 2D HSQC and HMBC correlations confirmed all carbon and proton assignments (Fig. S3 and S4).

**TABLE 1:** NMR assignments for HMAQ-7

Position	δ_C_ type	δ_H_ type (J in Hz)	HMBC^*a*^
1	NH	11.52 (br, 1H, NH)	
2	148.39, C		5, 8′
3	114.78, C		1′, 8′
4	176.94, CO		5, 7, 8′
4a	123.02, C		5, 6
5	125.51, CH	8.07 (d, 1H, J = 8.1, H5)	6, 7
6	123.34, CH	7.26 (dd, 1H, J = 7.9, J = 7.3, H6)	7, 8
7	118.09, CH	7.59 (dd, 1H, J = 7.9, J = 7.2, H7)	5, 6
8	131.55, CH	7.51 (d, 1H, J = 7.2, H8)	5, 6
8a	139.57, C		5, 6, 7
1′	35.33, CH_2_	3.39 (d, J = 3.5, 2H, H1′)	2
2′	124.78, CH	5.52 (dt, 1H, J = 5.1 & 2.3, H2′)	1′, 3′, 4′
3′	133.34, CH	5.53 (dt, 1H, J = 5.1 & 2.3, H3′)	1′, 2′, 4′, 5′
4′	31.89, CH_2_	1.97 (m, 2H, H4′)	3′, 5′, 6′
5′	31.31, CH_2_	1.27 (m, 2H, H5′)	4′, 6′, 7′
6′	21.97, CH_2_	1.22 (m, 2H, H6′)	4′, 5′, 7′
7′	14.18, CH_3_	0.82 (t, 3H, J = 7.4 H7′)	5′, 6′
8′	10.55, CH_3_	1.97 (s, 3H, H8′)	3, 2

^*a*^ Heteronuclear multiple bond correlations (HMBC) are from proton(s) to the stated carbon.

### Lliquid chromatography–high-resolution mass spectrometry (LC-HRMS) of HMAQ-7 isolated from *Burkholderia contaminans* MS14

To further support the structural assignments made for HMAQ-7, the compound was subjected to tandem mass spectrometry. Besides the protonated precursor ion of the native molecule (*m/z* 256.1698), 11 major fragment ions were identified in the spectrum (Fig. 1D). Eight of the detected fragment ion masses were consistent with cleavage of the C8 and C8′ methyl as well as sequential fragmentation of the alkyl chain up to the C2′ position (Table 2). One fragment had a mass consistent with the fragmentation of the alkyl chain (C2′ to C7′) as well as the carbonyl at C7 and methyl at C8′ (*m/z* 130.06512). Two additional fragments were observed with the loss of the C7 carbonyl, C8 and C8′ methyl, along with portions of the alkyl chain (*m/z* 144.08077 and *m/z* 118.06512). Fragmentation patterns provided supporting evidence of the covalent structure of HMAQ-7 and the alkenyl assignment for C2′ and C3′.

**TABLE 2:** Assignments of the tandem mass spectrometry data for the precursor parent ion for HMAQ-7 using HRMS

Formula	Fragments	Theoretical^*a*^ [M + H]^+^ (*m/z*)	Experimental^*a*^ [M + H]^+^ (*m/z*)	Mass error (ppm)*^b^*
C_17_H_21_N_1_O_1_	Parent—HMAQ-7	256.1696	256.1698	+0.85 ppm
C_15_H_15_NO	8′ [CH_3_]; 7′ [CH3]	226.12263	226.1224	−1.01 ppm
C_14_H_13_NO	8′ [CH_3_]; 6′, 7′ [CH2CH3]	212.10698	212.1071	+0.56 ppm
C_13_H_11_NO	8′ [CH_3_]; 5′ ,6′, 7′ [CH_2_CH_2_CH_3_]	198.09133	198.0913	−0.15 ppm
C_12_H_11_NO	3′, 4′, 5′, 6′, 7′ [CHCH_2_CH_2_CH_2_CH_3_]	186.09133	186.0914	+0.37 ppm
C_12_H_9_NO	8′ [CH_3_]; 4′, 5′, 6′, 7′ [CH_2_CH_2_CH_2_CH_3_]	184.07568	184.0758	+0.65 ppm
C_11_H_10_NO	2′, 3′, 4′, 5′, 6′, 7′ [CHCHCH_2_CH_2_CH_2_CH_3_]	173.08351	173.0835	−0.05 ppm
C_10_H_7_NO	8′ [CH_3_]; 2′, 3′, 4′, 5′, 6′, 7′ [CHCHCH_2_CH_2_CH_2_CH_3_]	158.06003	158.0600	−0.18 ppm
C_10_H_9_N	8′ [CH_3_]; 3, 4 [COC]; 4′, 5′, 6′, 7′ [CH_2_CH_2_CH_2_CH_3_]	144.08077	144.0808	+0.20 ppm
C_9_H_7_N	8′ [CH_3_]; 4 [CO]; 2′, 3′, 4′, 5′, 6′, 7′ [CHCHCH_2_CH_2_CH_2_CH_3_]	130.06512	130.0652	+0.61 ppm
C_8_H_7_N	8′ [CH_3_]; 3, 4 [COC]; 2′, 3′, 4′, 5′, 6′, 7′ [CHCHCH_2_CH_2_CH_2_CH_3_]	118.06512	118.0651	−0.16 ppm

^*a*^ Mass-to-charge ratio (*m*/*z*).

^*b*^ Parts per million (ppm).

### The antimicrobial activity of the isolated HMAQ-7 compound

A minimum inhibitory concentration (MIC) assay was used to evaluate HMAQ-7 inhibitory activity against one gram-negative bacterium, seven gram-positive bacteria, and three fungal species (Table 3). HMAQ-7 did not display any low micromolar inhibitory activity, and the lowest MICs observed were against the gram-positive bacterium *Clavibacter michiganesis* at 31.3 µg/mL and the mold *Aspergillus niger* at 62.5 µg/mL (Table 3). Despite the limited inhibitory activity of HMAQ-7, the compound was capable of inducing effects on the macro-agglutination phenotypes of several of the tested organisms. More precisely, the compound either prevented or engendered aberrations in the cellular clumping observed within the microtiter wells at the minimal effective concentration (MEC). These specific defects were induced by HMAQ-7 at low micromolar concentrations against all tested gram-positive bacterial strains with the exception of *Streptococcus mutans*. A noticeable difference in the floccular presentation was also observed against the fungal species tested at concentrations ranging between 15.6 and 31.3 µg/ml.

**TABLE 3:** Antimicrobial activity and growth defects induced by HMAQ-7

Target	Strain	MIC/MEC^*a*^ (µg/mL)	Biofilm response^*b*^
*Erwinia amylovora*	2029	500/125	NT
*Bacillus subtilis*	BS34a	>500/2.0	**↓** (≥10)**^*c*^**
*Micrococcus luteus*	ATCC Cohn 272	>500/2.0	NT
*Clavibacter michiganesis* subsp*. michiganesis*	Lu-01	31.3/3.9	NT
*Staphylococcus aureus*	TCH1516	>500/7.8	ND
*Staphylococcus haemolyticus*	MW-01	>500/7.8	**↓** (≥3.9)
*Streptococcus mutans*	JH1140	>500/>500	ND
*Saccharomyces cerevisiae*	DGY6	>500/31.3	NT
*Aspergillus niger*	ATCC 16888	62.5/15.6	NT
*Geotrichum candidum*	F-260	250/15.6	NT

^*a*^ Minimum effective concentration (MEC) is defined as the lowest concentration to show an observable growth difference from solvent control.

^*b*^ Qualitative effect of biofilm formation in the presence of HMAQ-7 at 24 h incubation. NT = not tested, ND = no effect detected, ↓ = inhibitory response. Effective concentration ranges are in parentheses.

^*c*^ Reported value is from standing culture experiment.

Of the 11 organisms utilized in the study, *Staphylococcus haemolyticus* exhibited the greatest sensitivity to HMAQ-7 in regard to its effect on the clumping morphology. We investigated whether these phenotypic effects corresponded to changes in bacterial growth by incubating *S. haemolyticus* with HMAQ-7 at 4× and 8× MEC in a shaking culture. Colony-forming unit (CFU) enumerations were performed at time points corresponding to the cultures lag phase (0–1 h), its early-to-mid log phase (2–4 h), and at stationary/early death phase (24 h). Enumerated CFUs of treated and control samples did not show a statistically significant difference in cell density (Fig. S5). These data would suggest that aggregation defects observed may not be a function of the compound’s toxicity but rather due to potential augmentations in cellular physiology and/or behavior.

Given these early experimental results, it became necessary to ascertain whether this morphological response corresponded to any clinically relevant phenotypes. The fact that HMAQ-7 prevents clumping is intriguing and suggests that it might be interfering with bacterial adhesion or aggregation mechanisms. Since bacteria tend to form biofilms at the bottom of wells, where they are embedded in a self-produced extracellular matrix that adheres to the interior surface, it seemed probable that interference of the aggregation morphology likely correlates to interference with related virulence factors such as biofilm formation.

### The anti-biofilm activities of HMAQ-7 against *S. haemolyticus* MW-01

**Fig 2:**
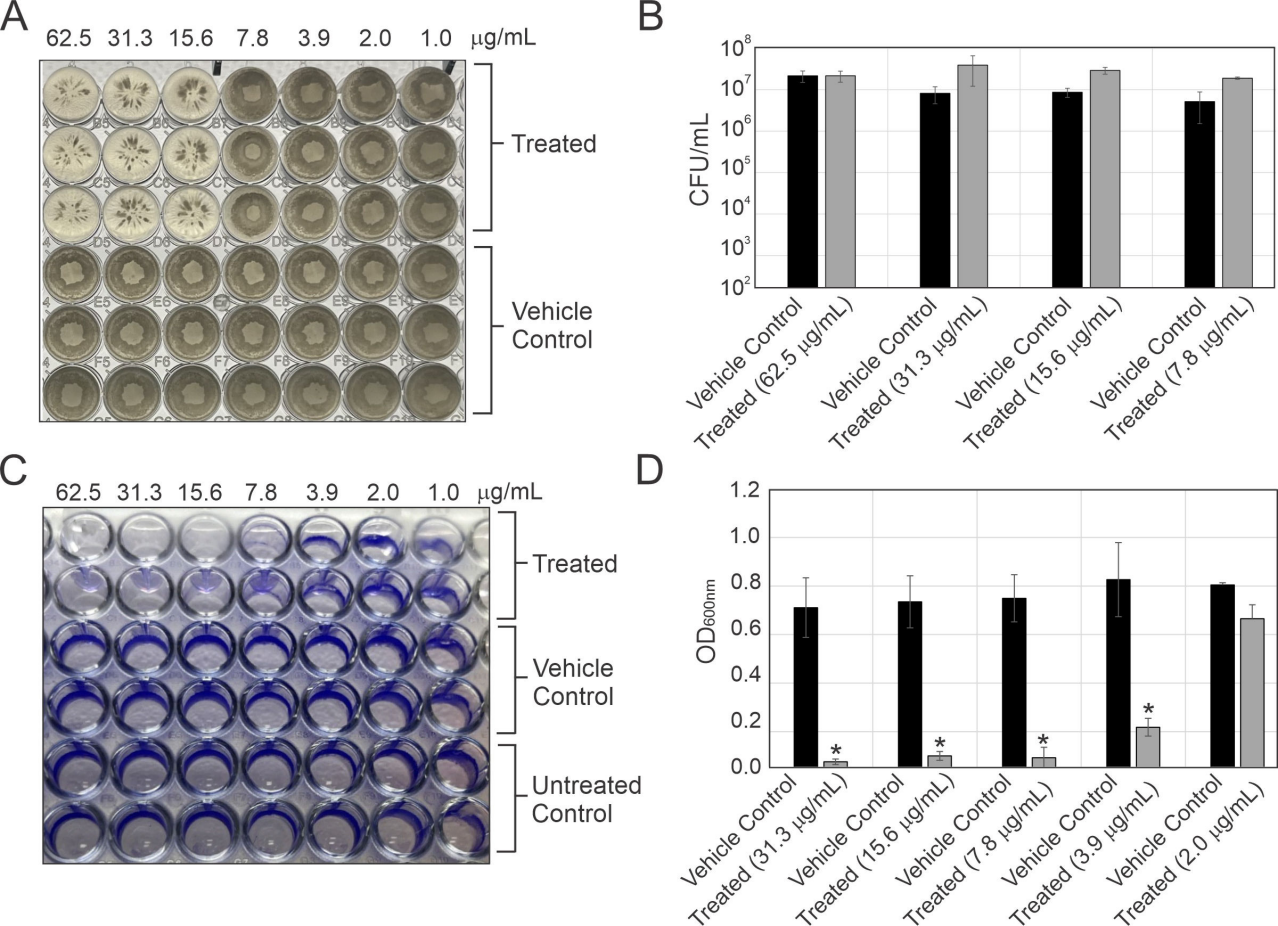
The isolated HMAQ-7′s effects on *S. haemolyticus* MW-01. (**A**) A qualitative observation of the effect that varying HMAQ-7 concentrations have on growth morphology after 24 h of incubation. Rows 1 to 3 show the treatment group, whereas rows 4 to 6 show the solvent control group. (**B**) Comparison of the CFUs between treatment and solvent control groups. Measurements were performed by vigorously mixing the cells in each well by pipetting before serial dilutions and colony numeration. Each bar represents a duplicate of triplicate experiments (*n* = 6 wells per condition). No statistically significant differences between the groups were observed. (**C**) Qualitative observation of the effect of varying HMAQ-7 concentrations on biofilm formation. The untreated control wells were visually identical to solvent control. (**D**) Quantification of biofilm density via OD_600_ measurements of the crystal violet extraction solution between the treatment and solvent control groups after 24 h of incubation. Each bar represents a duplicate of triplicate measurements (*n* = 6 wells per condition). Each of the representative treatment and solvent control samples was statistically significant (*P* < 0.05).

Pronounced differences in growth morphology were observed for *S. haemolyticus*, which had an MEC of 7.8 µg/mL. At 24 h of incubation, the growth of *S. haemolyticus* on the bottom of the treated wells showed a noticeable distortion in the aggregation phenotype (Fig. 2A). The aggregates appeared as desultory striations along the bottom of the wells instead of cohesive wide-bodied washers. Consistent with our growth experiments in shaking culture, this distortion did not appear to correspond with a substantial change in CFU/mL compared with the solvent control (Fig. 2B). As a phenomenon, the autoaggregation observed in the bottom of these wells is often highly correlated to biofilm formation (49). As was directly observed following crystal violet staining, the treated cells had an observable reduction in biofilm formation compared with the solvent control, which was qualitatively identical to the untreated group (Fig. 2C). These effects were also quantified through optical density measurements (OD_600_) (Fig. 2D). At a concentration of 3.9 µg/mL of HMAQ-7, which was below the observable minimum effective concentration, a statistically significant (*P* < 0.05) reduction in biofilm formation following exposure to HMAQ-7 was observed. The OD_600_ measurements of the crystal violet extraction solution (Fig. 2D) were approximately 0.2 for the treated versus 0.8 for the untreated group.

Since HMAQ-7 had no antibacterial activity against *S. haemolyticus* but rather inhibited its biofilm formation at low micromolar levels, we next asked if modulation in biofilm synthesis is observable in other bacterial species. Besides *S. haemolyticus*, *Bacillus subtilis* also displayed very interesting phenotypic changes in response to HMAQ-7. Given its status as a model organism, *B. subtilis* was used throughout the rest of the investigation to study the biological properties of HMAQ-7, including its potential effects on niche-specific phenotypes such as pellicle formation and sporulation.

### Phenotypic response of *B. subtilis* to HMAQ-7 exposure

**Fig 3:**
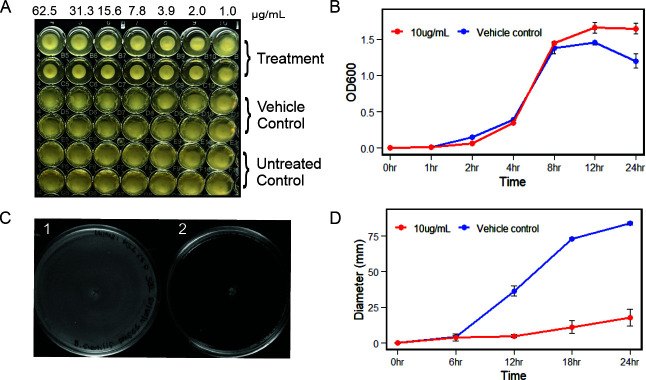
HMAQ-7 effects on growth and motility phenotypes in *B. subtilis*. (**A**) Qualitative observation of the effect that varying HMAQ-7 concentrations have on growth morphology/turbidity after 24 h of incubation. Rows 1 and 2 show the treatment group, rows 3 and 4 show vehicle control, and rows 5–6 show growth control. (**B**) Comparison of the growth rate when exposed to 5× MEC of HMAQ-7 or vehicle control at OD_600_. Each point represents experimental duplicates performed on separate days and technical duplicates (*n* = 4). (**C**) Swim motility assay performed with *B. subtilis*. Plate 1 shows no exposure to HMAQ-7, and plate 2 shows exposure to 5× MEC of HMAQ-7. Plate 2 showed a dramatic visual change in motility at 24 h. (**D**) Quantitation of *B. subtilis* swim diameter over a 24 h period. Each point represents experimental duplicates performed on separate days and technical duplicates (*n* = 4).

In the MIC/MEC assay for *B. subtilis*, a noticeable difference in flocculant morphology was apparent in the treated wells, with a low MEC of 2 µg/mL (Fig. 3A). Curiously, this change in morphology was also accompanied by a detectable reduction in the treated wells' turbidity. We initially suspected that the reduced opaqueness was due to a decrease in cell density; however, exposure to HMAQ-7 at 5× MEC (10 µg/mL, Table 3) did not correspond with any discernible change in either biomass or growth rates (Fig. 3B). Although cell viability remained unaffected during exposure, we hypothesized that the phenotype could be reflecting an effect on cellular locomotion. Turbidity is a well-established identifier of bacterial motility (50); thus, we employed the use of a classic swim motility assay using low-density agar containing 5× MEC of HMAQ-7 to assess the question. Compared with vehicle control, *B. subtilis* displayed complete suppression of its motility over a 24 h period when exposed to HMAQ-7 (Fig. 3C and D).

**Fig 4:**
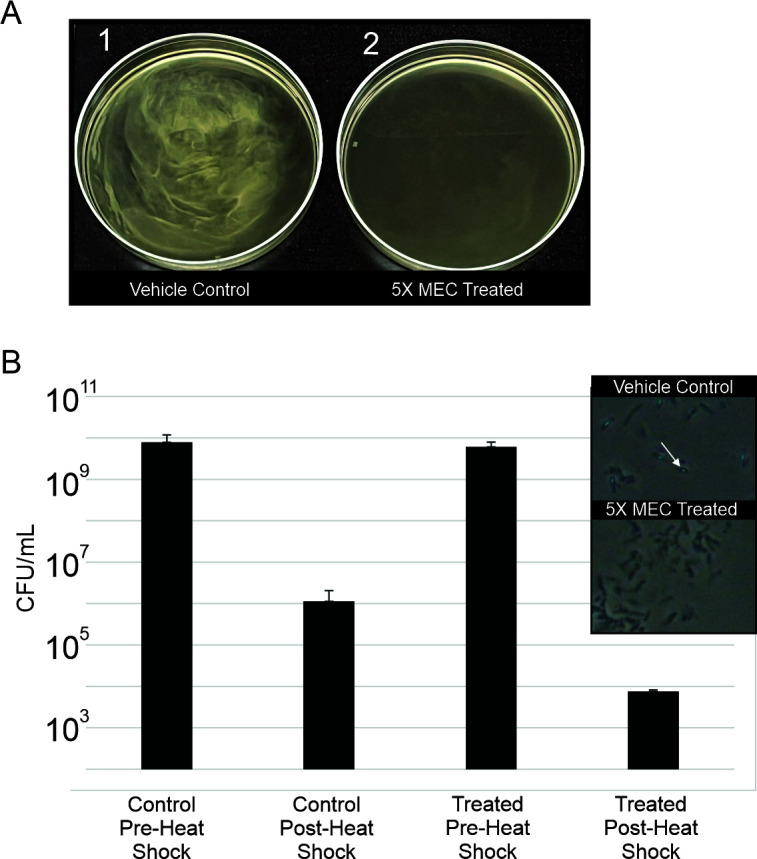
HMAQ-7 effects pellicle formation and spore formation in *B. subtilis*. (**A**) Qualitative observation of *B. subtilis* pellicle formation in standing culture after exposure to 5× MEC of HMAQ-7. Pellicle formation is absent in the 5× MEC of HMAQ-7 treated sample. (**B**) A 2-log reduction in cell density post-heat shock was observed in samples exposed to 5× MEC of HMAQ-7 at 24 h. An inset image shows a visual reduction in phase bright spores (see arrow) following exposure to HMAQ-7.

We then queried as to whether the turbidity could also be associated with a change in pellicle formation. Unlike most species of bacteria that produce biofilms, species such as those belonging to *Bacillus* do not form solid matrixes, but rather loose, low-density aggregates referred to as pellicles (floating biofilm) (51). Considering the previously established effects of HMAQ-7 to impede biofilm synthesis in *S. haemolyticus*, it seemed prudent to also investigate the effect in *B. subtilis*. As it turned out, besides effects on morphology and motility, exposure of *B. subtilis* to HMAQ-7 at 5× MEC resulted in clear inhibition of pellicle formation in standing culture (Fig. 4A).

Pellicle formation and sporulation are coregulated events in *B. subtilis* via the Spo0A transcriptional regulator, with matrix biosynthesis often serving as an inducer for spore formation under non-starvation conditions (52, 53). For this reason, we became curious as to whether the observed pellicle reductions seen here corresponded with a decrease in sporulation. We opted to measure this potential effect indirectly by measuring culture susceptibility to heat shock when exposed to the alkaloid. Indeed, *B. subtilis* incubated with 5× MEC of HMAQ-7 in standing culture showed not only an empirical difference in the presentation of its pellicle but in fact displayed a significant 2-log reduction in cell density post-heat shock (Fig. 4B). Microscope-based imaging also confirmed the reduction in heat-stable spore formation, with a noticeable reduction in spores after 24 h of exposure to HMAQ-7.

To better understand the global transcriptional response, *B. subtilis* exposed to HMAQ-7 was compared with untreated control to further our understanding of the global mechanisms behind the observed phenotypic changes.

### Transcriptional response of *B. subtilis* to HMAQ-7 exposure

Transcriptomics of *B. subtilis* 34A standing cultures identified 4,407 genes of which 1,869 showed significant differential expression (Table S1). Nearly one-third of all genes within the transcriptome (1,327 in total) showed highly significant differences (*P* < 0.001), and 542 genes showed moderate differences (0.001 < *P* < 0.05). The sheer volume of differentially expressed genes necessitated the use of an enrichment analysis that would highlight specific biological pathways and/or processes and structures that were being affected.

**Fig 5:**
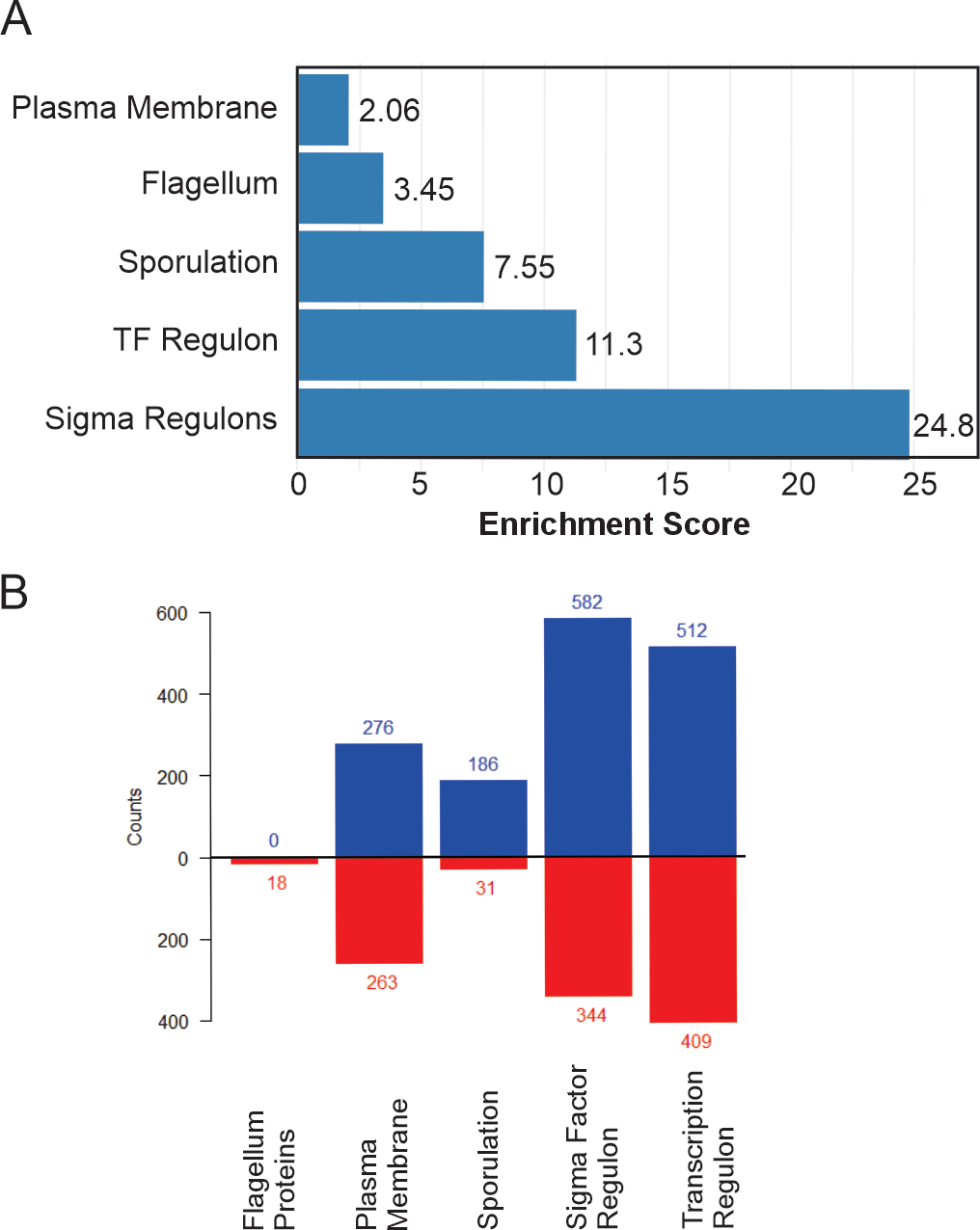
Effects of HMAQ-7 on transcriptional gene expression in *B. subtilis* 34A. (**A**) Results of non-directional enrichment analysis showing enrichment scores and corresponding enrichment categories. (**B**) Comparison of upregulated gene counts between control (blue) and treated (red) within each enrichment category. Total gene counts are displayed at the top of each bar.

Through the use of BioCyc’s omics dashboard, a non-directional enrichment analysis was performed with *B. subtilis* RNA-seq data. The query identified five enrichment categories wherein a significant number of genes within each were either over- or underrepresented: flagellum, plasma membrane, sigma factor (SF) regulon, sporulation, and transcription factor (TF) regulon. Among these, the SF regulon, TF regulon, and sporulation categories displayed the greatest enrichment, with scores of 24.8, 11.3, and 7.55, respectively (Fig. 5A and B). This augmentation of these categories is consistent with the diverse phenotypic responses seen previously, suggesting that quinolone induces direct and/or indirect effects on cellular regulatory elements.

When separated into its constituent regulons, we found that the SF regulon category experienced significant modulation in five key subsystems: sigG, sigF, sigE, sigK, and sigD, with sigGFEK regulon systems intimately tied to sporulation/germination. After tabulating the total number of genes with sizable differential expression (log_2_ fold change, ±1.5) across each of these systems, we found that 85% (318 of 376) of them were involved in sporulation/germination and that nearly all of these genes were downregulated in the treated group (Fig. S6). This near-ubiquitous downregulation of systematic processes was also observed in genes relevant to biofilm formation, including *sipW*, *tapA*, and the entirety of the *eps* operon excluding *epsC*.

Given the dynamic range of effects across variable species, we then asked what effects, if any, HMAQ-7 might actuate within complex multispecies biofilms.

### Exposure of complex biofilms to HMAQ-7

**Fig 6:**
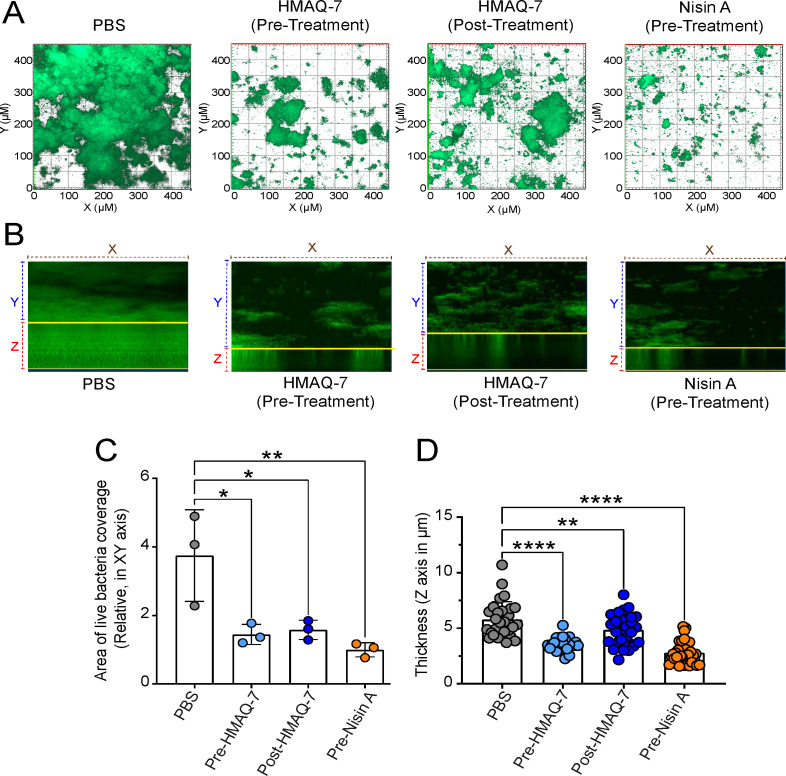
Disruption of oral biofilm formation by HMAQ-7. (**A**) Saliva from six healthy individuals was utilized to generate a multispecies biofilm in a well plate. The biofilm’s live/dead stain was qualitatively assessed following treatment with PBS, 50 µg/mL of nisin, or 50 µg/mL of HMAQ-7. Both pre-treatment with nisin or HMAQ-7 and post-treatment with HMAQ-7 were assessed to evaluate the efficacy in preventing oral biofilm formation and disrupting preformed biofilms, respectively. (**B**) Representative images demonstrate a reduction in the thickness of the multispecies biofilm following treatment with HMAQ-7 or nisin. The biofilm thickness was measured at 0.15 µm intervals using Z-stacks from the bottom (lowest signal intensity) to the top (highest signal intensity) of live cells (green). (**C**) Quantitative analysis of biofilm area (*XY*) showing pre-treatment with nisin or HMAQ-7 and post-treatment with HMAQ-7 led to a considerable decrease in the biofilm area. The area coverage, normalized to nisin treatment, was quantified from 10 images per condition, with each dot representing the average green coverage. (**D**) Quantitative analysis of biofilm thickness showing pre-treatment with nisin or HMAQ-7 and post-treatment with HMAQ-7 resulted in a significant reduction in biofilm thickness. Each dot represents one image. Data were obtained using custom Fiji (ImageJ) software. Statistical significance is denoted as *P* < 0.05 (*), *P* < 0.01 (**), and *P* < 0.0001 (***).

Oral species are well-documented to produce complex biofilms. Thus, we used a well-established protocol to evaluate the effects HMAQ-7 exerts on saliva-derived multispecies biofilms as described previously (54); 50 µg/mL HMAQ-7 would be added to either an oral biofilm propagated over 24 h without pre-exposure to the compound (post-treatment) or a biofilm generated in the presence of HMAQ-7 over the same time period (pre-treatment). The peptide antibiotic nisin at 50 µg/mL was used as a positive control under both experimental conditions, with PBS serving as a negative control. In these experiments, both nisin and HMAQ-7 had a statistically significant reduction in both biofilm coverage and thickness under both pre-treated and post-treated conditions (Fig. 6). These results provide strong evidence that HMAQ-7 not only suppresses biofilm formation but also possesses distinct properties that reduce the size of existing biofilms.

## DISCUSSION

This study constitutes one of the very few investigations into both the structural chemistry of 2-alkyl-4(1H)-quinolone metabolites produced by *Burkholderia* spp. and their effects on microbial physiology. Depoorter et al. (24) reported that the genus *Burkholderia* produces a structurally diverse group of these 4AQ metabolites and noted the need for furthering our understanding of their biological role. *B. contaminans* MS14 produces the quinolone metabolite HMAQ-7 in sufficient amounts that enable isolation and purification (Fig. 1B), thereby allowing for a more robust examination with regard to its structure and function. In this study, we have identified potent antibiofilm properties of HMAQ-7 against *S. haemolyticus*, which is one of the leading causes of nosocomial infections. The treatment of these infections is difficult due to its ability to form a robust biofilm (55, 56). We have further demonstrated its antibiofilm properties against a saliva-derived multispecies biofilm using a method previously reported (54).

Piochon et al. (28) synthesized the compound, named HMAQ1, with the same chemical composition as HMAQ-7. They reported low micromolar inhibitory activity against *B. subtilis* and other gram-positive bacterial species, whereas HMAQ-7 showed no such inhibition in growth against *B. subtilis* and other bacteria (Table 3). The lack of observed inhibitory activity against a large panel of organisms would suggest that the striking difference in the reported inhibitory activity by Piochon et al. is not due to differences in bacterial strains or species tested. The MEC values observed in our study are within the range of the MIC values they reported. Possibly, the MICs reported in this study are possibly being mistakenly reported for the observed MEC values. The biological activity observed for HMAQ-7 was clearly not inhibitory in nature following colony numeration, but rather an induction of drastic phenotypic changes.

The myriad effects that HMAQ-7 exerts on biologically diverse species like *S. haemlyoticus* and *B. subtilis* strongly indicate a dynamic role for this molecule ecologically. With respect to the compound’s biofilm-modulating properties, it may be hypothesized that *B. contaminans* is utilizing HMAQ-7 to prevent matrix formation and disrupt established biofilms in order to help promote its colonization in a multitude of environments. Biofilm formation is a well-known and documented virulence mechanism for bacterial and fungal pathogens (57–59). The isolated HMAQ-7 had limited inhibitory activity against the panel of bacterial and fungal species tested in this study (Table 3). Our findings demonstrate that the ability of HMAQ-7 to disrupt bacterial biofilms extends beyond monospecies biofilms, encompassing intricate multispecies biofilms on a global scale. This indicates certainty that *Burkholderia* possesses an ability to disrupt the colonization mechanism of competitive species through the disaggregating effects of HMAQ-7 on heterospecifics.

The variegated function of HMAQ-7 paired with the observation that it is the product of a soil commensal raises several questions. Although it does make sense that bacteria like *B. contaminans* would evolve the facilities to produce antibiofilm compounds to thwart colonization against fellow soil species, it is surprising that these compounds exert such a significant antibiofilm response in species for which *B. contaminans* would have little contact with except under very limited scenarios. This could be explained in part by shared regulatory pathways between species used in this study and other related organisms that *B. contaminans* comes into contact with in its native environments. Furthermore, *B. contaminans* MS14, as well as many other isolated strains of *Burkholderia*, is well-documented to produce antibacterial and antifungal compounds (6, 46, 48, 60). These antimicrobials would presumably be more effective if the bacterium could disrupt an existing biofilm. Alternatively, it could be that the production of HMAQ-7 plays a significant role in subverting colonization attempts among species that experience attenuation in biofilm production in the presence of the alkaloid, thus better facilitating *B. contaminans'* own colonization of the same environment.

Several plant extracts known to contain alkaloids and structurally related secondary metabolites have been previously shown to exert antibiofilm effects on oral species (61–64). Several of these antibiofilm effects seen in plant-derived alkaloids are known to be due to interference with quorum sensing, efflux pumps, and the release of extracellular components necessary for proper biofilm formation (62, 64). It is possible that HMAQ-7 may possess similar properties and facilitate its inhibition through similar mode(s) of action. With that in mind, we cannot exclude the possibility that exposure to HMAQ-7 is broadly dysregulating in ways that are still unknown to us. Although additional experiments are needed to determine if HMAQ-7 or similar metabolites are aiding in colonization and establishing infection, the hypothesis does provide a possible mechanism for Bcc occurrence in later stages of lung infection. Regardless of the precise reasons for the observed effects on biofilm, it does appear that the isolated HMAQ does modulate the functionality of potential competitor species and is able to inhibit and disrupt biofilm formation in ways that may be advantageous to *Burkholderia* during colonization in soil and potentially other clinically relevant environments.

It is hard to know from available data if the effects observed are necessarily the result of a single mode of action or multiple. With that said, we do clearly see that HMAQ-7 exposure induced drastic changes in global transcriptional response in *B. subtilis*, particularly with regard to its effects on other clinically relevant phenotypes such as sporulation and motility. Rather than the inhibition of these processes being mediated through the augmentation of one or a few genes, we can clearly identify broad fluctuations in expression across the entire transcriptome, including across several regulons. In the case of sporulation, we see clear agreement between near complete depletion of spore-associated gene expression in treated cells and the absence of sporulation experimentally. This same agreement was not observed with respect to cellular motility and membrane composition.

Enrichment analyses identified the repletion of gene expression in both gene groups associated with the plasma membrane and the flagellum proteins. Contrasting expression among sporulation genes, we see that exposure to HMAQ-7 clearly induces positive expression of genes encoding flagellum proteins as well as those controlled by sigD in treated cells. Genes associated with the sigD regulon (involved in cellular motility) experienced near-global upregulation in response to HMAQ-7 exposure, including most genes in the *che* operon and all genes in both the *fli* and *flg* operons. Given that earlier experimental data showed a negation of motility induced by HMAQ-7, this discordance between gene expression and present behavior would seem paradoxical prima facie. It could be hypothesized that the loss of motility is attributable to a concomitant loss of proton motive force. This assumption is supported by the significant changes in transcriptional regulation of proteins associated with the membrane, suggesting that HMAQ-7 is targeting the membrane directly or, at the very least, inducing an adaptive change in its composition. Further support of this claim may also come from the clear enrichment of several membrane transport genes, several of which are under the direct control of CcpA, a regulator involved in carbon utilization and catabolism (38) (Fig. S6). This observation is of great interest, as it would not only suggest changes in the membrane but would also indicate a nutrient adaptation response. This is evidenced by the apparent upregulation of genes such as those involved in pulcherrimin biosynthesis and myoinositol synthesis in treated cultures, components that would provide alternative methods of acquiring necessary metals as well as carbon sources.

No specific cellular target has been identified in this study, but the data do support changes in the cellular membrane based on the upregulation of membrane transporter genes. Given the wide array of phenotypic changes and changes in gene expression, more than one target likely exists for HMAQ-7. To this end, the acquisition of high-quality metabolomics and proteomics information may help elucidate the biological activity of HMAQ-7 and related metabolites. Although this study raises many questions about how this molecule functions, the study provides a solid foundation for future work investigating the mechanism of action in which the compound acts to trigger the observed antibiofilm properties. In addition, the findings in this study support efforts to further understand the potential HMAQ-7 has on the virulence of *Burkholderia* spp., as well as studies on HMAQ-7 use in animal systems to determine its ability to mitigate infection and disease by disrupting biofilm formation.

## MATERIALS AND METHODS

### General experimental procedures

NMR analysis was performed as described previously by our group (47). Approximately 5.0 mg of the isolated product was dissolved in 600 µL of DMSO-d6, and the NMR data were collected on a Bruker Avance 600 DRX NMR spectrometer operating at a proton frequency of 600 MHz. The 1D proton and C13 data were collected, and the resonances were assigned according to standard methods. A 1D proton NMR was collected for J coupling measurements between H2 and H3 in CDCl3 using a Bruker Avance III-HD 850 MHz NMR spectrometer. The 2D HSQC and HMBC NMR experiments were used to clarify and confirm proton and carbon assignments. The HSQC and HMBC NMR experiments were collected at 25°C. The spectral sweep widths for HSQC and HMBC NMR experiments were 11.25 ppm in the proton dimensions and 165 ppm for the carbon dimension and centered at the water peak at about 3.3 ppm. The HSQC and HMBC NMR experiments were collected with 1024 and 192 complex points in the direct and indirect dimensions, respectively. Topspin version 4.14 was used in analyzing the data.

### Mass confirmation of HMAQ-7 with liquid-chromatography mass spectrometry

Analysis of HMAQ-7 was performed using a LC-HRMS system comprised of a Nexera X2 MP Ultrahigh-Performance Liquid Chromatography (UHPLC) system from Shimadzu (Kyoto, Japan) coupled to an OrbiTrap Fusion Tribrid HRMS system from ThermoFisher (Waltham, MA USA). Operational control of the LC system was performed using the Shimadzu LabSolutions software (Ver. 5.82). Data acquisition and operational control of the HRMS system was performed using Xcalibur (Ver. 4.3.73.11). The chromatographic peak and spectral review for HMAQ-7 was performed using the Xcalibur-based Qual Browser application. HMAQ-7 stock solution was prepared by dissolving a 5 mg quantity of HPLC-purified material in a 5 mL volume of acetonitrile to yield a final concentration of 1 mg/mL. To ensure complete dissolution of all solids, the stock solution was vortex-mixed for 5 min, which resulted in a completely clear stock solution. A 10 µL volume of the HMAQ-7 stock solution was diluted in a 990 µL volume of a solution consisting of ACN/water/FA (5/95/0.1, vol/vol/vol) to produce a HMAQ-7 sample at a final concentration of 10 µg/mL for analysis. A 10 µL volume was injected into the LC-HRMS system for analysis.

LC separations were performed using mobile phase (MP) solutions consisting of 0.1% formic acid (FA) in water (MPA) and 0.1% FA in ACN (MPB), and a needle wash solution consisting of ACN:water (1:1, vol:vol). The chromatographic method included column heating at 40°C, autosampler tray chilling at 15°C, a mobile phase flow rate of 0.080 mL/min, and a gradient elution program specified as follows: 0–2.0 min, 20% MPB; 2.0–14.0 min, 20%–100% MPB; 14.0–17.0 min, 100% MPB; 17.0–17.2 min, 100%–20% MPB; and 17.2–20.0 min, 20% MPB. The overall cycle time for a single injection was approximately 20.5 min.

The ion source and global method parameters for the OrbiTrap Fusion HRMS system were specified as follows: application mode, “small molecule”; method duration, 19.5 min; ion source type, heated electrospray (H-ESI) ion source; spray voltage, static; positive ion voltage, +3,500 V; sheath gas (Arb), 5; aux gas (Arb), 2; sweep gas (Arb), 0; ion transfer tube temperature, +275°C; vaporizer temperature, +20°C; infusion mode, liquid chromatography; expected peak width, 60 s; default charge state, +1; and, internal mass calibration, “Easy-IC.” The PRM-specific MS (1) scan parameters for the HRMS system were specified as follows: detector type, Orbitrap; Orbitrap resolution, 120,000; mass range, normal; use quadrupole isolation, true; scan range, *m/z* 75–*m/z* 375; RF lens, 60%; AGC target, custom; normalized AGC target, 50%; maximum injection time mode, custom; maximum injection time, 50 ms; microscans, 1; data type, profile; polarity, positive; source fragmentation, disabled; and use Easy-IC, true. The PRM-specific tMS (2) scan parameters for the HRMS system were specified as follows: MSn level, 2; multiplex ions, false; isolation mode, quadrupole; isolation window (*m/z*), *m/z* 1.6; activation type, HCD; stepped collision energy, true; ±HCD collision energy (%), 5%; detector type, Orbitrap; Orbitrap resolution, 15,000; mass range, normal; scan range mode, auto; RF lens, 60%; AGC target, standard; maximum injection time mode, auto; microscans, 1; data type, centroid; polarity, positive; source fragmentation, disabled; use Easy-IC, true; and loop control, all. The PRM-based mass list tables below list the precursor ion for HMAQ-7 that was selected for MS2 spectral scanning (Table 2).

Optima LC/MS-grade water, acetonitrile (ACN), and FA were each purchased from Fisher Scientific (Waltham, MA, USA). The analytical and guard columns used for this study included a 3 μm Luna C18(2) (100 Å; 150 mm × 1 mm; P/N: 00 F-4251-A0) and a SecurityGuard Cartridge C18 (4 mm × 2 mm; P/N: AJ0-4286) which were each purchased from Phenomenex (Torrance, CA, USA). All other solvents or chemicals were ACS grade or better and were purchased from Sigma-Aldrich (St. Louis, MO) or VWR (Radnor, PA).

### Purification of HMAQ-7

Isolation of HMAQ-7 was performed as previously reported for occidiofungin with some modifications (44). The producing strain, *B. contaminans* MS14, was grown in a 1 L volume of modified minimal M9 media with the glucose substituted for asparagine (1 g/L). The final pH of the medium was adjusted to 6.9. A 10% inoculum with an OD_600_ between 0.6 and 0.8 was made before incubating the culture at 28°C for 3 to 4 days. The inoculum was prepared using 1 L of the culture media seeded with a 1 mL glycerol stock of MS14 and was shaken at 200 RPM while incubating at 28°C. Cell-free supernatant (CFS) was made by filtering the culture media across a column packed with Amberlite XAD1180N resin, which binds the HMAQ-7 compound. The resin was then rinsed with water before HMAQ-7 was eluted from the resin using an 80:20 isopropanol:water solution. This cell-free supernatant was then passed through a 0.2 µm filter before drying under vacuum. The resultant dried material was then rinsed with a 200 mL volume of acetonitrile, which extracts the HMAQ-7 compound from insoluble material. The acetonitrile fraction was then dried again under vacuum and then suspended in a sufficient volume of 80:20 acetonitrile:water (vol/vol) with 0.1% trifluoroacetic acid to re-solubilize all of the material. This preparation was then subjected to reverse phase chromatography using a 5-μm C18 SinChrom ODS-BP (10 mm × 250 mm) preparatory column using a DuoFlow HPLC (Bio-Rad Laboratories, Hercules, California) at a mobile phase flow rate of 3 mL/min at room temperature. LC separations were performed using mobile phase (MP) solutions consisting of 10 mM ammonium formate in water (MPA) and ACN (MPB). The chromatography method consisted of a gradient elution program specified as follows: 0–5.0 min, 10% MPB; 5.0–32.0 min, 10%–75% MPB; 32.0–37.0 min, 75% MPB; 37.0 min, 75%–10% MPB; and, 37.0–42.0 min, 10% MPB. The fraction of interest eluted at 75% MPB and was analyzed via MALDI-TOF for confirmation of the desired product. A Shimadzu/Kratos matrix-assisted laser desorption ionization–time of flight (MALDI-TOF) mass spectrometer was used to confirm the expected mass isolated from HPLC fractions using a 1:1 mix of the HPLC eluent and α-cyano-4-hydroxycinnamic acid matrix (10 mg/mL in 50:50 acetonitrile:water with 0.1% TFA). A 1 µL sample was spotted on the MALDI-TOF target plate and ionized using a laser strength between 20% and 30% with no mass suppression in the positive linear mode or reflective mode. After mass confirmation, the purified sample was freeze-dried and weighed on an analytical scale (Adventurer Pro AV114C; Ohaus Corporation, USA). The powder would then be suspended in dimethyl sulfoxide for bioassays

### HMAQ-7 physical properties

HMAQ-7’s physical properties were as follows: amorphous off white/tan powder; UV (water) λmax 220; LC-HRMS *m/z* 256.1698 (calculated for C_17_H_21_NO); see Table 1.

### Bacterial strains and growth conditions

*Erwinia amylovora* 2029 and *Clavibacter michiganesis* subsp. *michiganesis* Lu-01 were cultured on nutrient broth yeast extract (NBY) medium as described previously (65). *Bacillus subtilis* BS34a, *Micrococcus luteus* ATCC Cohn 272, *Staphylococcus aureus* TCH1516, and *Staphylococcus haemolyticus* MW-01 were cultured on Todd-Hewitt yeast extract (THyex) medium as mentioned previously (66). *Saccharomyces cerevisiae* DGY6BY, *Aspergillus niger* ATCC 16888, and *Geotrichum candidum* F-260 were cultured on yeast-peptone-dextrose (YPD) medium or potato-dextrose-agar (PDA) (67). *Burkholderia contaminans* MS14 was cultured on either NBY medium (44) or in modified M9 media containing L-asparagine (1 g/L L-asparagine).

### Growth morphology observations and colony numeration

Early investigations into the biological effects of HMAQ-7 on indicator bacterial species involved a modification to the microdilution broth assay reported in the Clinical and Laboratory Standards Institute (CLSI) M07-A8, CLSI M27-A3, and CLSI M38-A2 susceptibility testing of bacteria, yeasts, and filamentous fungi, respectively. Briefly, a fresh plate(s) (16–24 h incubation for bacteria and 24–48 h for fungi) of a given test species is inoculated into its appropriate growth medium broth to an OD_600_ of 0.2. This inoculum was then diluted such that the final inoculum with appropriate media (Nutrient broth yeast extract for plant bacterial pathogens, potato dextrose broth for plant fungal pathogens, TSB + 0.25% sucrose for aerobic gram-positive bacteria) in each well of a 96-well plate was between 2 and 8 × 10^5^ CFU/mL for the bacterial tests and 2 and 8 × 10^4^ CFU/mL for the fungal tests. Purified HMAQ-7 was dissolved in 100% DMSO at a stock concentration of 10 mg/mL. A series of 2-fold microdilutions of HMAQ-7 (500–1 µg/mL) was performed in a round bottom 96-well plate. A 1:1 (vol/vol) addition of inoculum to media with a final well volume of 200 µL was then sealed with parafilm and incubated at 37˚C (plant pathogens at 28˚C). The MIC was determined as the minimum concentration that had no visible growth in the well for 24 h (plant pathogens for 48 h). The minimum effective concentration was determined as the lowest concentration that had an observable difference from the control sample. Specifically for the *Staphylococcus haemolyticus* assays, the biological effects would first be evaluated qualitatively based on the floccular morphology as observed at the bottom of the wells. To determine the total volume of planktonic cells in each well, the contents were thoroughly mixed via pipette and aliquoted after the initial incubation period. Aliquots were serially diluted and then spread-plated to determine colony-forming units. Plates were incubated for 24 h for viable cells enumeration. All reported measurements are from three independent experiments with duplicate samples.

Growth curve study was performed using the modified protocol described in Mengxin et al (68). Briefly, a fresh plate of *Staphylococcus haemolyticus* MW-01 was incubated between 16 and 24 h at 37°C. A tube containing 10 mL of newly made THyex broth would be inoculated to an initial OD_600_ of 0.1 before being placed in a shaking incubator. The tube was allowed to incubate at 37°C and 240 rpm until reaching an OD_600_ 0.5–0.6 at which time a 100-fold dilution would then be performed on the inoculum, resulting in a 2.3 × 10^5^ CFU/mL cell density. HMAQ-7 was solubilized into a 10 mg/mL stock solution using 100% DMSO. Enough of the drug would be added to the tube such that the final working concentration for the treatment tubes was 4× or 8× MEC. In addition to the treatment tubes, tubes containing an equivalent volume of DMSO (<1% total volume) would be used as vehicle controls. Treatment and vehicle tubes would be made in duplicate. Both sets of tubes would be incubated at 37°C and 240 rpm. A 100 µL aliquot would be taken from each tube at 0, 1, 2, 4, and 24 h time points. Each aliquot would undergo serial dilution for CFU determination. All plates were incubated at 37°C for 24 h before CFU determination. All curves represent a duplicate of duplicate experiments (*n* = 4).

### Pellicle experiments and spore calculation

Pellicle experiments were set up using a modified method described in Pisithkul et al. 2019 (69). Briefly, a fresh plate of indicator species is inoculated into tryptic soy broth (TSB) containing 0.25% sucrose to an OD_600_ of between 0.2 and 0.3. A 100-fold dilution is then performed on this inoculum into 10 mL of identical media containing either 10 µg/mL of HMAQ-7 or an equivalent volume of DMSO to serve as vehicle control. The contents of the tubes are thoroughly vortexed before being deposited into sterile petri dishes (VWR polystyrene, 100 × 15 mm) and incubated at 37°C for 24 to 48 h. Pellicle formation is evaluated qualitatively and photographed. Experiments are performed in parallel duplicates of duplicates (*n* = 4). For *B. subtilis* 34A, after 24 h incubation, the contents of these plates would be pipetted into pre-weighted falcon tubes and centrifuged at 13,400 × *g* for 5 min at 20°C. The supernatants are subsequently decanted, and the pellets are resuspended in 1× PBS to a final volume of 2 mL and vortexed. In addition, a 10-fold serial dilution of the tubes is performed down to 10^7^ and plated to ascertain cell density. After this, the tubes containing the pellets are heat shocked at 80°C for 20 min. After heating, serial dilutions of the contents are performed again, plated, and stored at 37°C for 24 h before CFU enumeration. Qualitative evaluation of the spore content was achieved through the use of a confocal microscope with phase contrast settings at a total magnification of 400×, with 2 µL either treated or control cultures being spotted onto a glass microscope slide.

### RNA-seq library preparation

Samples were sent to Zymo Research for Total RNA-Seq Service. Libraries were constructed from the total RNA sample. Libraries were prepared using the Zymo-Seq RiboFree Total RNA Library Prep Kit (Cat # R3000) according to the manufacturer’s instruction manual v1.3.1. Briefly, RNA was reverse transcribed into cDNA, which was followed by ribosomal RNA depletion. After that, a partial P7 adapter sequence was ligated at the 3′ end of cDNAs, followed by second-strand synthesis and partial P5 adapter ligation to the 5′ end of the double-stranded DNAs. Finally, libraries were amplified to incorporate full-length adapters under the following conditions: initial denaturation at 95°C for 10 min; 10–16 cycles of denaturation at 95°C for 30 s, annealing at 60°C for 30 s, and extension at 72°C for 60 s; and final extension at 72°C for 7 min. Successful library construction was confirmed with Agilent’s D1000 ScreenTape Assay on TapeStation. RNA-seq libraries were sequenced on an Illumina NovaSeq to a sequencing depth of at least 30 million read pairs (150 bp paired-end sequencing) per sample.

### RNA-seq data bioinformatics analysis

The Zymo Research RNA-Seq pipeline was originally adapted from nf-core/rnaseq pipeline v1.4.2 (70). The pipelines were built using Nextflow (71). Briefly, quality control of raw reads was carried out using FastQC v0.11.9. Adapter and low-quality sequences were trimmed from raw reads using Trim Galore! v0.6.6. Trimmed reads were aligned to the reference genome using STAR v2.6.1d (72). BAM file filtering and indexing were carried out using SAMtools v1.9 (73). RNA-seq library quality control was implemented using RSeQC v4.0.0 (74) and QualiMap v2.2.2-dev (75). Duplicate reads were marked using Picard tools v2.23.9 (76). Library complexity was estimated using Preseq v2.0.3 (77). Duplication rate quality control was performed using dupRadar v1.18.0 (78). Reads overlapping with exons were assigned to genes using featureCounts v2.0.1 (79). Classification of rRNA genes/exons and their reads were based on annotations and RepeatMasker rRNA tracks from the UCSC genome browser when applicable. Differential gene expression analysis was completed Ver 3.0 using DESeq2 v1.28.0 (80). Functional enrichment analysis was achieved using g:Profiler python API v1.0.0 (81). Quality control and analysis results plots were visualized using MultiQC v1.9 (82).

### BioCyc navigation and enrichment query

The RNA-seq data were reformatted into a tab-delimited file type. Data were uploaded and converted into a smart table. Due to the limited curation of the BS34A strain, the genes were mapped to corresponding orthologs in the better-curated *B. subtilis* 168 strain, which would also serve as the reference genome for all analyses. Non-directional enrichment analyses were performed through the pathway tools omics dashboard. For the enrichment analysis, the parameters were set for a Fisher exact statistical test with a significance threshold set to *P* < 0.05 accompanied by a Bonferroni multiple hypothesis correction.

### Biofilm inhibition assays

Effects on biofilm formation were quantified via OD_600_ measurements. After a 24 h incubation period, a 96-well plate would undergo a modified washing protocol as described in O’Toole et al. (83). Briefly, the plate is removed from the incubator, and the liquid contents of each well are carefully removed by pipette. The side of each well is then vigorously mixed with ddH_2_O 3–4 times per well. A 1% crystal violet solution is then added to each well and then allowed to incubate for 15 min after which the crystal violet is then pipetted out and each well rinsed again 3–4 times with ddH_2_O. The plate is positioned upside down on a paper towel to allow for drying. Once the wells were fully dry, qualitative features of the biofilms were photographed. For quantitation, 200 µL of 100% DMSO was added to each well with a pipette and then transferred to a new 96-well plate. A 200 µL volume of a given well was diluted 3-fold in a cuvette with DMSO, and the OD was measured. OD_600_ readings between 0.000 and 0.900 were considered acceptable (not requiring additional dilutions). All measurements were performed in a minimum of a duplicate of triplicate experiments.

Saliva-derived biofilms were established in 24-well glass-bottomed SensoPlates (Greiner Bio-One, Monroe, NC, USA) as described previously (49, 84). The formed biofilms were then challenged with either nisin or HMAQ-7 at 50 µg/mL for 30 min (post-biofilm treatment), and PBS was used as a negative control. The cells used to seed the biofilm plates were also pretreated with HMAQ-7 at 50 µg/mL for 30 min (pre-biofilm treatment). Nisin and HMAQ-7 were removed from the biofilm by washing twice with PBS. Following the manufacturer’s instructions, the biofilm cells were labeled with Filmtracer LIVE/DEAD Biofilm Viability Kit (Invitrogen, USA). The stains were removed by washing twice with PBS, and then the biofilms were fixed using 1.6% paraformaldehyde (PFA). PFA was removed by PBS washing, and then Fluoromount-G Mounting Medium (Invitrogen, USA) was added to thin coverslips before flipping over each biofilm sample. The samples were left to mount overnight in the dark at room temperature. Microscopy and imaging were carried out as follows: all biofilm images were acquired by a Zeiss LSM 800 with the Plan Apochromat objectives featuring Zeiss 20× (Plan Apochromat; 20×/0.8). 3D biofilm images were constructed using ZEN software (Zeiss). The mean intensity of pixels for green (Syto-9; live) and red (PI; dead) signals was measured within the biofilm areas of each image, with the slices containing the highest respective signal intensity using custom Fiji (ImageJ) (85). All quantifications and statistical calculations are based on 10–15 images and 5–10 stacks for each triplicate.

The thickness of biofilms was acquired with intervals of 0.15 µm between each slice. Z-stacks were defined from the bottom with the lowest signal intensity to the top with the highest signal intensity of live cells (green). Representative images for thickness (Z-axis size, reported in μm) were created using the Volume Viewer plugin in Fiji with a consistent scale of 1.3 for all conditions. For each biological replicate, approximately 10 images from various spots were captured, and statistical analyses were performed based on the number of images. Three biological replicates were included for the multispecies biofilm for each condition/treatment.

### Statistical analysis

Statistically significant differences between treatment and solvent controls in terms of CFU/mL and biofilm production were determined via a two-sample *t*-test assuming unequal variance. A statistically significant difference was defined as one having a minimum measured *P* < 0.05 after appropriate Bonferroni correction.

## Data Availability

The RNA-seq data have been deposited in the NCBI Sequence Read Archive (SRA). The BioProject accession number is PRJNA1236414.

## SUPPLEMENTAL MATERIAL

Supplemental Tables and Figures msphere.01081-24-s0001.docx Tables S1 to S3; Fig. S1 to S6.

ASM does not own the copyrights to Supplemental Material that may be linked to, or accessed through, an article. The authors have granted ASM a non-exclusive, world-wide license to publish the Supplemental Material files. Please contact the corresponding author directly for reuse.
