# Supplementary material for: Antibiofilm properties of 4-hydroxy-3-methyl-2-alkenylquinoline, a novel Burkholderia-derived alkaloid
Source: mSphere. 2025 May 8;10(5):e01081-24. doi: 10.1128/msphere.01081-24 (PMC12108052; doi:10.1128/msphere.01081-24)
Supplement: Supplemental Tables and Figures — Tables S1 to S3; Fig. S1 to S6. [file msphere.01081-24-s0001.docx]

**Supporting Information**

**Antibiofilm Properties of 4-hydroxy-3-methyl-2-alkenylquinoline, a Novel *Burkholderia*-Derived Alkaloid**

McKinley D. Williams, Taylor R. Sweeney, Sabrina Trieu, Ravi Orugunty, Abdelahhad Barbour, Fereshteh Younesi, Michael Glogauer, Nopakorn Hansanant, Ronald Shin, Shi-En Lu, Kevin Cao, Abraham Tenorio, Sigmund J. Haidacher, Anthony M. Haag, Thomas D. Horvath, Leif Smith^#^

^a^Department of Biology, Texas A&M University, College Station, TX, United States

^b^Antimicrobial Division, Sano Chemicals Inc., Bryan, TX, United States,

^c^Faculty of Dentistry, University of Toronto, Toronto, Ontario, Canada

^d^Central Alabama High-Field NMR Facility, Structural Biology Shared Facility, Cancer Center, University of Alabama at Birmingham, Birmingham, Alabama, USA

^e^Department of Biochemistry, Molecular Biology, Entomology and Plant Pathology, Mississippi State University, Mississippi State, Mississippi, USA

^f^Department of Pathology and Immunology, Baylor College of Medicine, Houston, Texas, USA.

^g^Texas Children's Microbiome Center, Texas Children's Hospital, Houston, Texas, USA.

^h^Department of Pharmacy Practice and Translational Research, College of Pharmacy, University of Houston, Houston, Texas, USA

^*^Address correspondence to Leif Smith, [jsmith@bio.tamu.edu](mailto:jsmith@bio.tamu.edu)

**Table S1.** Transcriptomic statistics for *B. subtilis* culture.

**Table S2.** Top 50 downregulated genes in *B. subtilis* treated cultures following exposure to HMAQ-7 during comparative transcriptomic analysis.

**Table S3.** Top 50 upregulated genes in *B. subtilis* treated cultures following exposure to HMAQ-7 during comparative transcriptomic analysis.

**Figure S1.** ^13^C NMR spectrum of HMAQ-7 in DMSO-d6 (600 MHz).

**Figure S2.** ^1^H NMR spectrum of HMAQ-7 H2’ and H3’ coupling in CDCl3 (850 MHz).

**Figure S3.** HSQC spectrum of HMAQ-7 in DMSO-d6 (600 MHz).

**Figure S4.** HMBC spectrum of HMAQ-7 in DMSO-d6 (600 MHz).

**Figure S5.** Effect of HMAQ-7 on *Staphylococcus haemolyticus* MW-01 growth

**Figure S6.** Additional analyses of enrichment transcriptomic changes in *B. subtilis*.

**Table S1.** Transcriptomic statistics for *B. subtilis* culture

| Total # Genes Detected | 4407 |
| --- | --- |
| # of Downregulated Genes in Treated | 1050 |
| # of Upregulated Genes in Treated | 819 |
| # of genes with highly significant DE (p<0.001) | 1327 |
| # of genes with moderately significant DE (0.001<p<0.05) | 542 |
| % Differentially expressed genes | 42.4% |
| Ratio of Downregulated to Upregulated genes | 1.28:1 |

**Table S2.** Top 50 downregulated genes in *B. subtilis* treated cultures following exposure to HMAQ-7 during comparative transcriptomic analysis.

| Gene Name | Function | Associated Enrichment Categories* | Fold Change | Adjusted P-value |
| --- | --- | --- | --- | --- |
| *yhzE1 (sscA)* | Spore assembly and germination protein | Sporulation/Sigma/TF | 14.5 | 6.57796E-20 |
| *cotF* | Spore coat protein | Sporulation/TF | 12.4 | 1.37096E-36 |
| *cmpA* | Cortex morphogenic protein A; represses cortex assembly until the successful initiation of spore coat assembly | Sporulation | 12.2 | 9.67629E-14 |
| *ykzV* | Unknown (Associated with Sporulation) | Sporulation | 12.1 | 1.7825E-119 |
| *cotNE* | Inner spore coat protein | Sporulation | 11.6 | 8.2342E-104 |
| *VV28_RS09090* | Unknown | NA | 11.5 | 3.81043E-50 |
| *VV28_RS19655* | Unknown | NA | 11.4 | 5.9298E-178 |
| *yhzE2* (*sscB*) | Spore and germination protein | Sporulation/Sigma/TF | 11.2 | 7.22395E-12 |
| *cotY* | Main structural component of the spore crust; necessary for the assembly of the spore crust, the outermost layer of the spore coat | Sporulation/Sigma/TF | 11.2 | 2.7225E-158 |
| *yqfT* | Unknown (Associated with Sporulation) | Sporulation | 11.0 | 2.84746E-81 |
| *yppG* | Spore coat protein | Sporulation | 10.9 | 1.22676E-48 |
| *spoVIF* | Required for spore coat assembly and resistance | Sporulation/Sigma/TF | 10.9 | 8.3287E-99 |
| *VV28_RS05475* | Unknown | NA | 10.8 | 2.65183E-39 |
| *cotZ* | Spore crust anchor protein; necessary for the assembly of the spore crust, the outermost layer of the spore coat | Sporulation/Sigma/TF | 10.8 | 2.297E-99 |
| *glnH* | Glutamine ABC transporter (binding protein) | TF/Plasma Membrane | 10.8 | 4.97431E-58 |
| *ydgB* | Unknown | NA | 10.6 | 6.98086E-26 |
| *ytcB* | Putative UDP-glucose epimerase (expressed late during sporulation in mother cell) | Sporulation | 10.6 | 6.89041E-70 |
| *gerPF* | Spore germination protein; facilitates access of nutrient germinants to their cognate germinant receptors in spores’ inner membrane | Sporulation/Sigma/TF | 10.6 | 7.81655E-67 |
| *ytzH* | Unknown (Expressed during sporulation) | Sporulation | 10.2 | 1.15147E-10 |
| *cotW* | Spore crust protein (insoluble fraction); involved in the formation of the spore crust | Sporulation/Sigma/TF | 10.2 | 2.2383E-119 |
| *yitB* | Phospho-adenylylsulfate sulfotransferase | NA | 10.2 | 2.72395E-52 |
| *yjcZ* | Unknown (Associated with sporulation) | Sporulation | 10.1 | 2.5711E-72 |
| *ytcA* | Similar to UDP-sugar dehydrogenase | NA | 10.1 | 2.39958E-84 |
| *cotX* | Spore crust protein (insoluble fraction); necessary for the assembly of the spore crust, the outermost layer of the spore coat | Sporulation/Sigma/TF | 10.0 | 2.8129E-138 |
| *cotV* | spore crust protein (insoluble fraction); involved in the formation of the spore crust | Sporulation/Sigma/TF | 10.0 | 2.9889E-138 |
| *VV28_RS21635* | Unknown | NA | 10.0 | 3.99323E-10 |
| *VV28_RS21500* | Unknown | NA | 10.0 | 9.79556E-36 |
| *VV28_RS06285* | Unknown | NA | 10.0 | 1.53273E-70 |
| *mmgA* | Degradative acetoacetyl-CoA thiolase | Sigma Regulon/TF regulon | 9.9 | 4.6021E-112 |
| *sspG* | Small acid-soluble spore protein (minor) | Sporulation/Sigma/TF | 9.9 | 9.17736E-33 |
| *cotG* | Outer spore coat protein | Sporulation/Sigma/TF | 9.9 | 3.3559E-99 |
| *sacT* | Transcriptional antiterminator for the sacP-sacA-ywdA operon | TF Regulon | 9.9 | 9.7937E-141 |
| *VV28_RS16455* | Unknown | NA | 9.8 | 1.93027E-16 |
| *yyaC* | Sporulation protein | Sporulation/Sigma | 9.8 | 2.99936E-82 |
| *sspO* | Small acid-soluble spore protein (minor) | Sporulation/Sigma | 9.7 | 7.02861E-39 |
| *tasA* | Major component of biofilm matrix; forms bundles of fibers | Sporulation/Sigma/TF | 9.7 | 2.5686E-117 |
| *gerQ* | Spore coat protein; is necessary for the proper localization of CwlJ | Sporulation/Sigma | 9.7 | 2.0712E-107 |
| *cotT* | Spore coat protein (inner) | Sporulation/Sigma/ TF | 9.7 | 1.09552E-82 |
| *cgeC* | Maturation of the outermost layer of the spore | Sporulation/Sigma/TF | 9.6 | 5.18905E-45 |
| *ytfJ (gerW)* | Germination protein | Sporulation/ Sigma | 9.6 | 3.97856E-90 |
| *sgpA* | Spore germination protein | Sporulation/TF | 9.6 | 4.6838E-107 |
| *VV28_RS20410* | Unknown | NA | 9.6 | 1.2325E-41 |
| *cysC (ylnC)* | Adenylyl-sulfate kinase | NA | 9.5 | 6.49201E-72 |
| *VV28_RS03925* | Unknown | NA | 9.5 | 2.52831E-71 |
| *VV28_RS03225* | Unknown | NA | 9.5 | 9.06256E-63 |
| *sspB* | Small acid-soluble spore protein (major beta-type SASP) | Sporulation/Sigma/TF | 9.4 | 3.9834E-104 |
| *yqfQ* | Late sporulation protein | Sporulation | 9.4 | 2.30712E-90 |
| *ymxH* | Sporulation protein | Sporulation | 9.4 | 1.64117E-62 |
| *mmgC* | Acyl-CoA dehydrogenase | Sporulation/Sigma/ TF | 9.4 | 1.08027E-85 |
| *cgeA* | Spore crust glycoprotein, anchors polysaccharides to the spore surface, maturation of the outermost layer of the spore | Sporulation/Sigma/TF | 9.3 | 1.92604E-75 |

*Enrichment categories are those depicted in Figure 5; Assignment based upon function and/or identification in query; NA indicates non-assignment.

**Table S3.** Top 50 upregulated genes in *B. subtilis* treated cultures following exposure to HMAQ-7 during comparative transcriptomic analysis.

| Gene Name | Function | Associated Enrichment Category* | Fold Change | Adjusted P-value |
| --- | --- | --- | --- | --- |
| *pchE* | Pyochelin synthetase (siderophore) | NA | 7.9 | 5.62941E-55 |
| *cypX* | cytochrome P450, cyclo-l-leucyl-l-leucyl dipeptide oxidase | TF | 7.7 | 8.35828E-60 |
| *pchR* | transcriptional repressor (MarR family); controls the expression of genes involved in pulcherriminic acid biosynthesis | TF | 7.6 | 1.10441E-52 |
| *gmuA* | glucomannan-specific permease of the phosphotransferase system; EIIA of the PTS | Sigma/TF | 7.6 | 6.47966E-74 |
| *gmuD* | phospho-beta-mannosidase | Sigma/TF | 7.4 | 2.05897E-73 |
| *pchC* | Thioesterase (Optimizes Biosynthesis of Pyochelin) | NA | 7.4 | 2.40079E-57 |
| *celB* | lichenan-specific permease of the phosphotransferase system, EIIC of the PTS | NA | 7.3 | 5.36079E-71 |
| *gmuB* | glucomannan-specific permease of the phosphotransferase system, EIIB of the PTS | Sigma/TF | 7.1 | 3.87635E-63 |
| *manA (yjdE)* | mannose-6-phosphate isomerase, required for proper cell wall synthesis (mutants lose rod shape) | NA | 6.6 | 6.99917E-59 |
| *gmuR* | transcriptional repressor (GntR family) of the gmuB-gmuA-gmuC-gmuD-gmuR-gmuE-gmuF-gmuG operon | Sigma/TF | 6.6 | 4.97431E-58 |
| *gmuE* | mannose kinase | Sigma/TF | 6.5 | 2.27548E-59 |
| *czcD* | cation exporter (antiporter); involved in the export of zinc in exchange for extracellular K+ and H+ | NA | 6.1 | 4.25716E-44 |
| *gmuG* | beta-1,4-mannanase | Sigma/TF | 5.9 | 9.99789E-53 |
| *treP* | trehalose permease of the phosphotransferase system; EIIBC of the PTS | Sigma/TF/Plasma Membrane | 5.6 | 4.87397E-46 |
| *ybeC (aimA)* | general amino acid importer; low affinity symporter for glutamate/H+, serine/H+, asparagine/H+, glycine[metabolite\|]/H+, diaminopropionic acid/H+, alanine/H+, and beta-alanine/H+ | Plasma Membrane | 5.6 | 7.19505E-36 |
| *copO* | metal (copper) efflux transporter | NA | 5.6 | 7.64361E-40 |
| *amtB* | ammonium transporter; required at low ammonium concentration | Sigma/TF/Plasma Membrane | 5.5 | 4.15004E-32 |
| *treC (treA)* | phospho-alpha-glucosidase | Sigma/TF | 5.4 | 2.19579E-35 |
| *rbsB* | ribose ABC transporter (binding protein) | Sigma/TF/Plasma Membrane | 5.1 | 6.61476E-31 |
| *msmR (melR)* | transcriptional regulator of the melR-melE-melD-melC-melA operon | Sigma/TF | 5.1 | 1.01571E-23 |
| *rbsC* | ribose ABC transporter (permease) | Sigma/TF/Plasma Membrane | 5.1 | 2.91855E-32 |
| *sdhB* | succinate dehydrogenase | Sigma/Plasma Membrane | 5.0 | 1.10992E-23 |
| *cimH* | transporter for citrate (proton symport) | TF/Plasma Membrane | 5.0 | 1.14098E-30 |
| *cadA* | cadmium transporting ATPase, resistance to cadmium | Sigma/Plasma Membrane | 4.9 | 1.3597E-34 |
| *bdhA (ydjL)* | acetoine/ butanediol dehydrogenase | NA | 4.9 | 6.63549E-29 |
| *ysmA* | similar to thioesterase | NA | 4.9 | 4.70131E-23 |
| *yybF* | similar to antibiotic resistance protein; Putative permease | Plasma Membrane | 4.9 | 1.70152E-26 |
| *tet(M)* | Mobile element that promotes tetracycline resistance | NA | 4.8 | 4.30443E-19 |
| *clpE* | AAA unfoldase, ATPase subunit of the ClpE-ClpP protease (class III stress gene) | Sigma/TF | 4.8 | 4.74291E-29 |
| *bglP* | beta-glucoside permease of the phosphotransferase system, EIIBCA of the PTS, Trigger enzyme, control of LicT activity | Sigma/TF/Plasma Membrane | 4.8 | 3.83238E-28 |
| *pxpG* | 5-oxoproline transporter | NA | 4.7 | 1.49533E-17 |
| *VV28_RS21600* | Unknown | NA | 4.7 | 4.23386E-29 |
| *yvcA* | required for complex colony development; Putative lipoprotein | NA | 4.7 | 2.14703E-18 |
| *sdhA* | succinate dehydrogenase (flavoprotein subunit) | Sigma/Plasma Membrane | 4.7 | 2.04669E-22 |
| *bglH* | phospho-beta-glucosidase | Sigma/TF | 4.7 | 3.6396E-25 |
| *yrdP (czcO)* | similar to flavin-containing monooxygenase, facilitates cation export via CzcD | Plasma Membrane | 4.7 | 1.77731E-21 |
| *ydbP* | similar to thioredoxin | Sigma | 4.6 | 2.72035E-26 |
| *pxpA* | subunit of ATP-dependent 5-oxoprolinase | NA | 4.5 | 1.68468E-29 |
| *iolF* | D-chiro-inositol transport protein | Sigma/TF/Plasma Membrane | 4.4 | 4.97266E-26 |
| *guaC* | GMP reductase | TF | 4.4 | 0.003589465 |
| *glnR* | transcriptional repressor (MerR family) of the glnR-glnA operon | Sigma | 4.4 | 2.56294E-19 |
| *iolG* | inositol 2-dehydrogenase | NA | 4.4 | 3.26017E-26 |
| *iolE* | 2-keto-myo-inositol dehydratase, dehydration of 2-keto-myo-inositol (2nd reaction) | Sigma/TF | 4.4 | 1.57233E-24 |
| *iolC* | formation of 2-deoxy-5-keto-gluconic acid-6-phosphate (5th reaction) | Sigma/TF | 4.4 | 1.42081E-21 |
| *yqjL* | general stress protein; putative hydrolase involved in oxidative stress resistance; important for survival at low temperature | Sigma | 4.3 | 6.06648E-22 |
| *iseA* | secreted inhibitor of cell wall endopeptidases; inhibits cell separation | Sigma/TF | 4.3 | 6.41608E-23 |
| *blt* | spermidine-efflux transporter | Sigma/TF/Plasma Membrane | 4.3 | 3.10431E-16 |
| *iolH* | unknown, may be involved in myo-inositol catabolism | Sigma/TF | 4.3 | 1.17569E-23 |
| *bdbA* | thiol-disulfide oxidoreductase | TF | 4.2 | 1.59966E-05 |
| *iolD* | 3D-(3,5/4)-trihydroxycyclohexane-1,2-dione hydrolase; involved in formation of 5-deoxy-D-glucuronic acid (3rd reaction) | Sigma/TF | 4.2 | 7.79383E-22 |

*Enrichment categories are those depicted in Figure 5; Assignment based upon function and/or identification in query; NA indicates non-assignment.


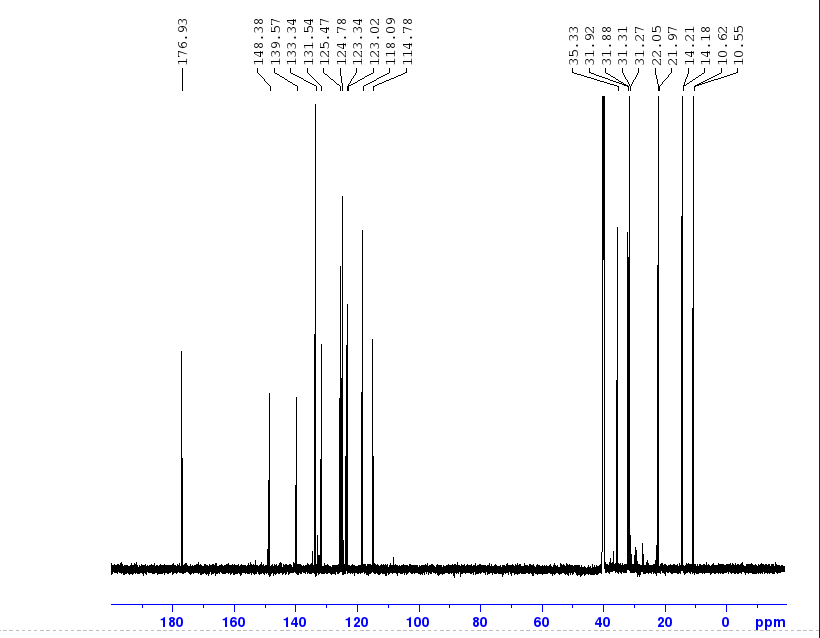


**Figure S1.** ^13^C NMR spectrum of HMAQ-7 in DMSO-d6 (600 MHz).


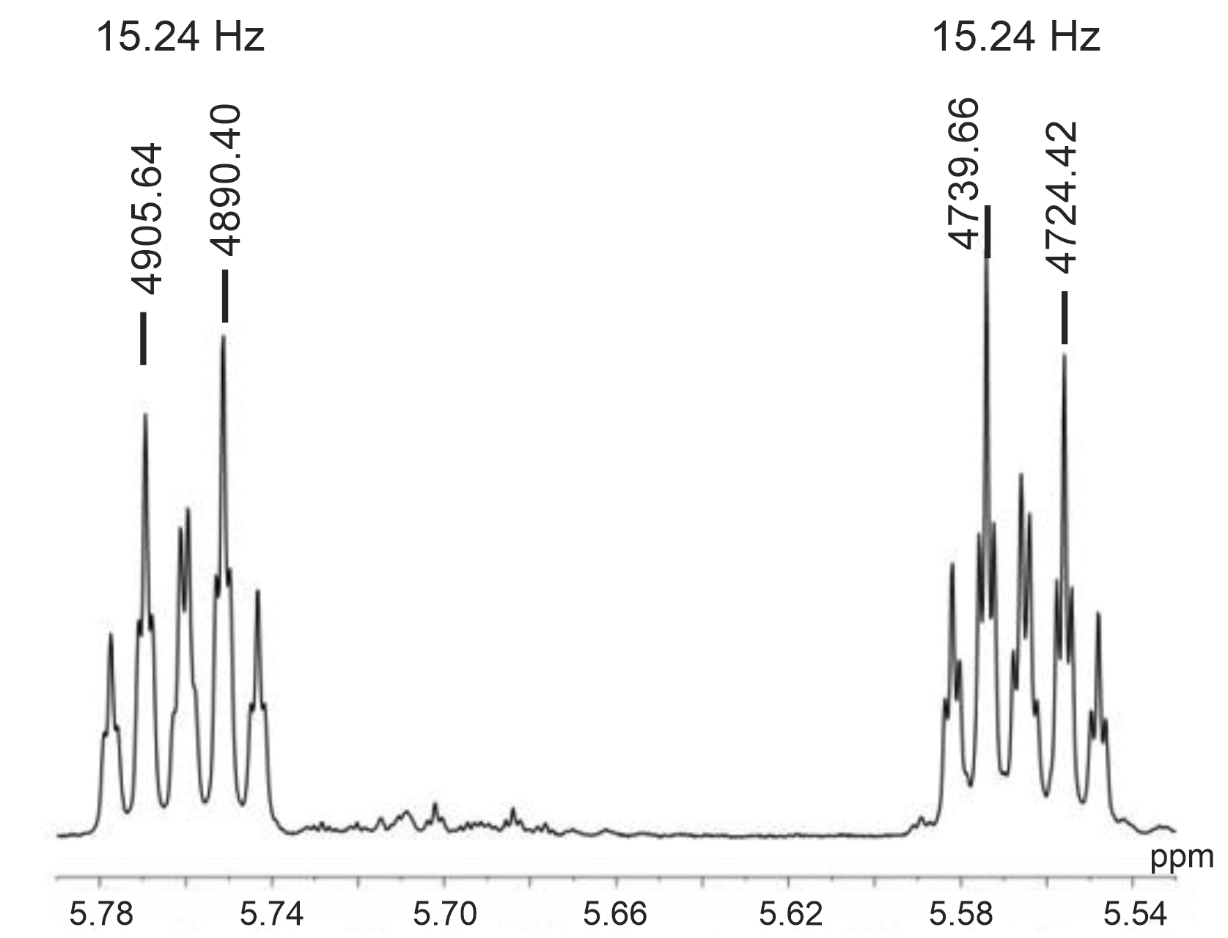


**Figure S2.** ^1^H NMR spectrum of HMAQ-7 H2’ and H3’ coupling in CDCl3 (850 MHz).


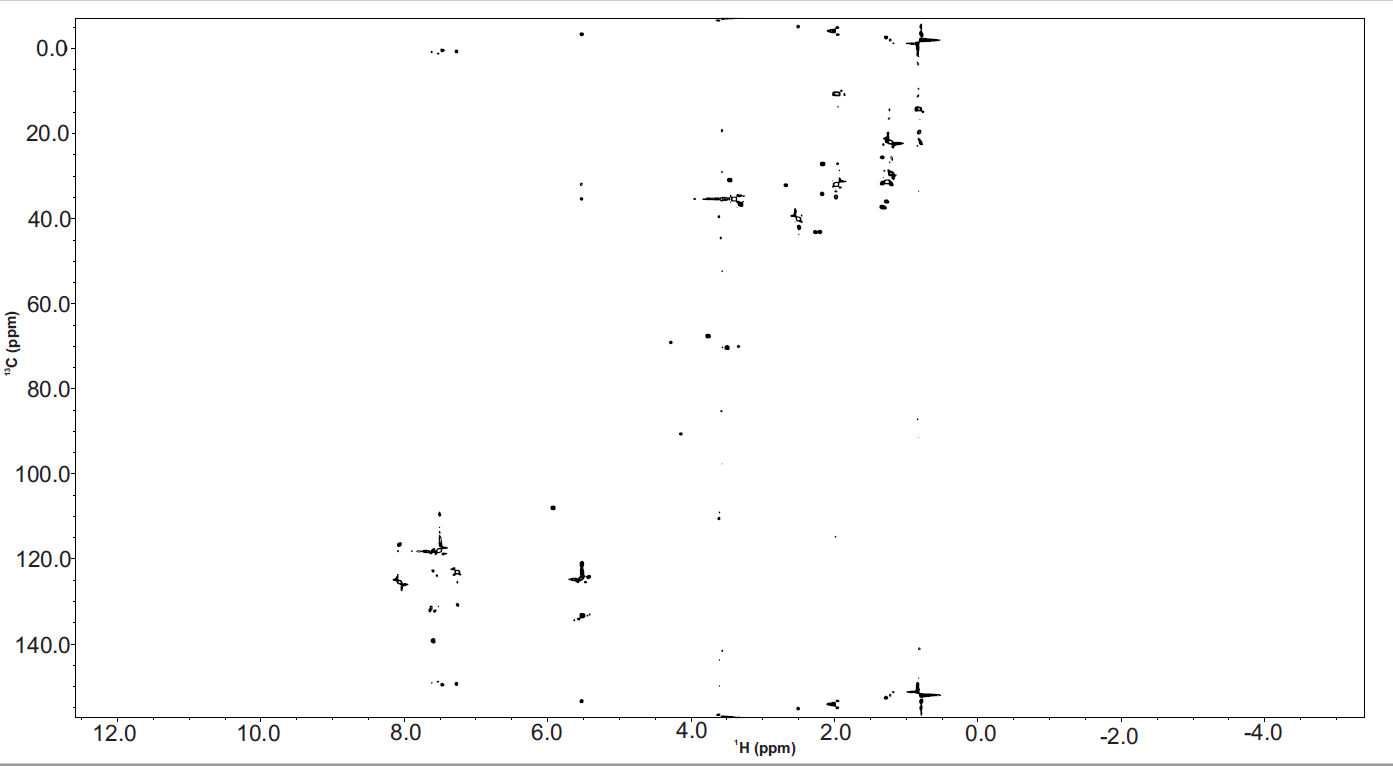


**Figure S3.** HSQC spectrum of HMAQ-7 in DMSO-d6 (600 MHz).


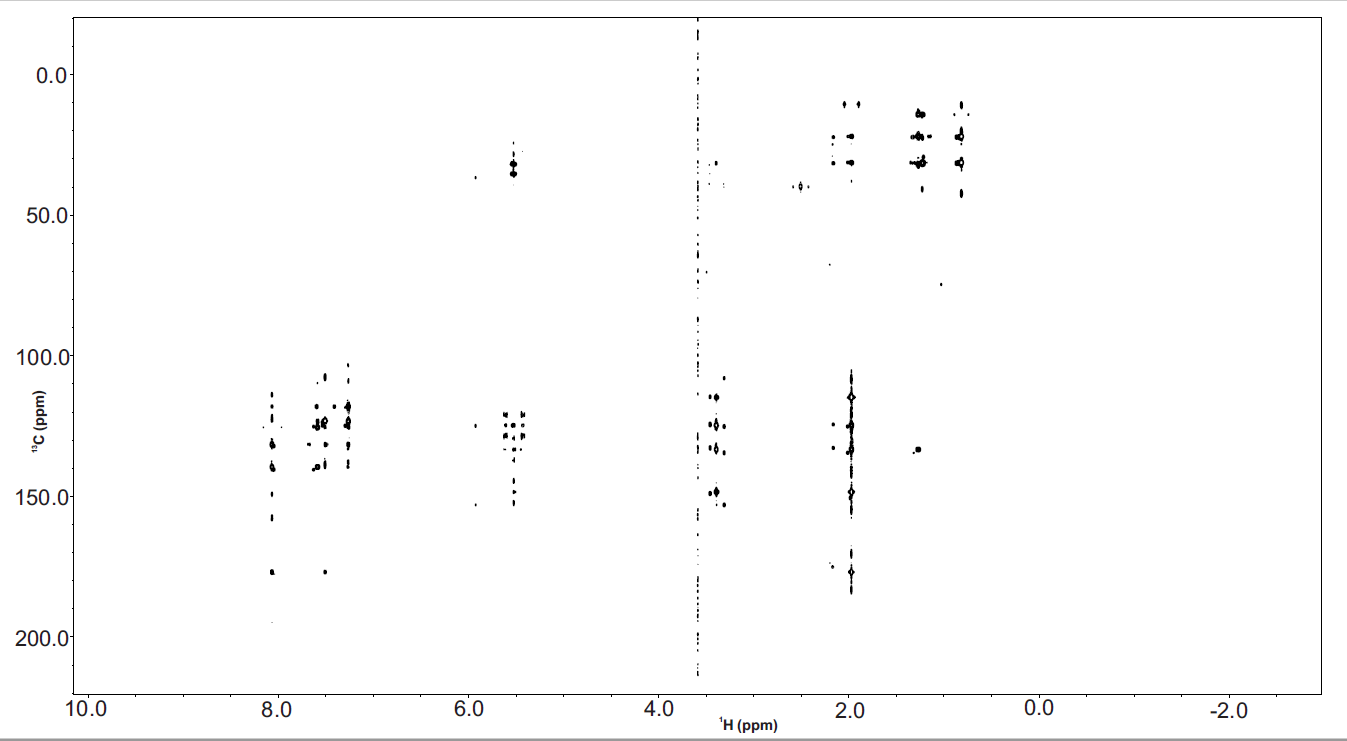


**Figure S4.** HMBC spectrum of HMAQ-7 in DMSO-d6 (600 MHz).

**Figure S5.** Cell forming units per mL of *Staphylococcus haemolyticus* MW-01 following exposure to HMAQ-7. Cell forming units were determined at 0 hours (lag phase), 2 hours (early log phase), 4 hours (log phase), and 24 hours (stationary phase). There was no statistical difference between the cell densities for each group at the specified time points.


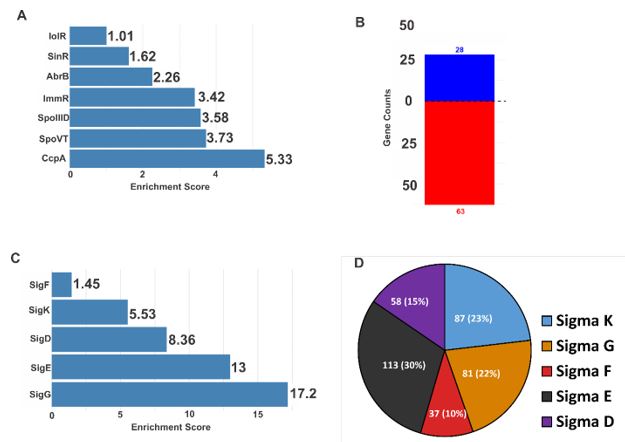


**Figure S6. Additional analyses of enrichment transcriptomic changes in *B. subtilis*. A.)** Result of a non-directional enrichment analysis showing enrichment scores and corresponding categories for transcription factor subsystems. **B**.) Comparison of upregulated gene counts between control (blue) and treated (red) in the abrB regulon. Total gene counts displayed at the top of each bar. **C**.) Result of a non-directional enrichment analysis showing enrichment scores and corresponding categories for differing sigma factor regulons. **D**.) Distribution of total genes across all enriched SF regulon categories. Corresponding percentages for each category are displayed in parentheses next to counts.
